# A *tps1Δ* persister-like state in *Saccharomyces cerevisiae* is regulated by *MKT1*

**DOI:** 10.1371/journal.pone.0233779

**Published:** 2020-05-29

**Authors:** Patrick A. Gibney, Anqi Chen, Ariel Schieler, Jonathan C. Chen, Yifan Xu, David G. Hendrickson, R. Scott McIsaac, Joshua D. Rabinowitz, David Botstein

**Affiliations:** 1 Lewis-Sigler Institute for Integrative Genomics, Princeton University, Princeton, New Jersey, United States of America; 2 Calico Life Sciences LLC, South San Francisco, California, United States of America; 3 Department of Food Science, Cornell University, Ithaca, New York, United States of America; 4 Department of Chemistry, Princeton University, Princeton, New Jersey, United States of America; University of Nebraska-Lincoln, UNITED STATES

## Abstract

Trehalose metabolism in yeast has been linked to a variety of phenotypes, including heat resistance, desiccation tolerance, carbon-source utilization, and sporulation. The relationships among the several phenotypes of mutants unable to synthesize trehalose are not understood, even though the pathway is highly conserved. One of these phenotypes is that *tps1Δ* strains cannot reportedly grow on media containing glucose or fructose, even when another carbon source they can use (e.g. galactose) is present. Here we corroborate the recent observation that a small fraction of yeast *tps1Δ* cells do grow on glucose, unlike the majority of the population. This is not due to a genetic alteration, but instead resembles the persister phenotype documented in many microorganisms and cancer cells undergoing lethal stress. We extend these observations to show that this phenomenon is glucose-specific, as it does not occur on another highly fermented carbon source, fructose. We further demonstrate that this phenomenon appears to be related to mitochondrial complex III function, but unrelated to inorganic phosphate levels in the cell, as had previously been suggested. Finally, we found that this phenomenon is specific to S288C-derived strains, and is the consequence of a variant in the *MKT1* gene.

## Introduction

Individuals within isogenic cultures can exhibit stochastic phenotypic variability that appears not to be genetically encoded. This phenotype spans organisms from bacteria to human cancer cells. In exponential phase bacterial cultures, a small subpopulation of cells can survive a variety of stressors, and have consequently been termed “persister cells” [1]. In *S*. *cerevisiae*, a similar phenomenon was observed within exponentially growing cells, and the small heat-resistant subpopulation was suggested to conform with the evolutionary biology concept of “bet hedging” [2]. Similarly, within tumor cells, and in tumor cell culture, a small fraction of individuals resistant to chemotherapeutic agents arise through non-genetic means [1,3]. Taken together, these phenomena share the common theme that small fractions of a population exhibit non-genetic variability in stress resistance. This is a major problem in medicine, where this can present as antibiotic resistance of microbial pathogens, or resistance to chemotherapy within tumors. How these phenomena are regulated remains unclear.

Trehalose metabolism is a highly conserved metabolic pathway that produces and degrades trehalose—a disaccharide of glucose [4]. While some organisms, including animals, have lost the ability to produce trehalose, there are species throughout all three domains of life that are able to both produce and break down trehalose [5]. Much of the work done to understand the role of trehalose metabolism has been undertaken in fungi, most often in *Saccharomyces cerevisiae* [6]. Trehalose metabolism has important implications for a wide variety of cellular processes in fungi, ranging from regulation of glycolysis to virulence [6–8]. Trehalose itself has been proposed to act as a storage carbohydrate, a molecular chaperone to counteract protein unfolding stress, or as a water substitute to protect cellular membranes and contents against desiccation [6,7]. Recent work suggests that trehalose itself does play a role in desiccation tolerance, though not other stress tolerances [9,10]. Despite decades of research, however, no model exists for the role of trehalose metabolism in the fungal cell that can explain all of the observed phenotypes associated with this pathway.

While it has been long known that disruption of the *TPS1* gene results in cells unable to grow on fermentable carbon sources such as glucose and/or fructose, in 2014 van Heerden *et al*. reported that a distinct subpopulation of cells in an isogenic *tps1Δ* culture is unexpectedly able to grow on glucose-containing media, and this subpopulation is not genetically determined [11–19]. They suggest that this division is related to stochastic fluctuations in intracellular inorganic phosphate levels that determine glycolytic dynamics, and that these cells can be detected based on their cytosolic pH. They further suggest that the role of trehalose metabolism is to provide proper phosphate balance in the cell. In this model, also proposed previously, the main role of trehalose metabolism is to liberate inorganic phosphate from trehalose-6-phosphate, allowing that phosphate to be used as a substrate for the glyceraldehyde-3-phosphate dehydrogenase (GAPDH) enzyme in glycolysis [13,16,20].

Here we independently validate that a small fraction of *tps1Δ* cells in a clonal population is able to grow on glucose. We also recapitulated that this fraction of cells does not represent true genetic suppressors of the *tps1Δ* glucose growth defect. While similar to the persister state, persister cells survive stressful conditions in a dormant state. As these cells both survive and grow while experiencing stress, in this case glucose, we are referring to them as persister-like cells. We further extend previous observations in a number of ways. We demonstrate that development of persister-like cells is glucose-specific, and does not occur with fructose—another fermentable sugar long known to result in growth inhibition of *tps1Δ* [11,12,14–16,18,19,21,22] Further, the number of persister-like cells can be enhanced or reduced by media components (peptones and/or nitrogen source), and is enhanced by inactivation of respiratory complex III or cytochrome c. We also present multiple lines of evidence that this phenomenon is not related to inorganic phosphate release, but is instead a consequence of a S288C-specific allele of the *MKT1* gene. To our knowledge this is the first example of a genetic perturbation that causes a persister-like phenotype in eukaryotes.

## Results

### *tps1Δ* persister-like cells occur in S288C-derived strains grown on glucose

**Fig 1:**
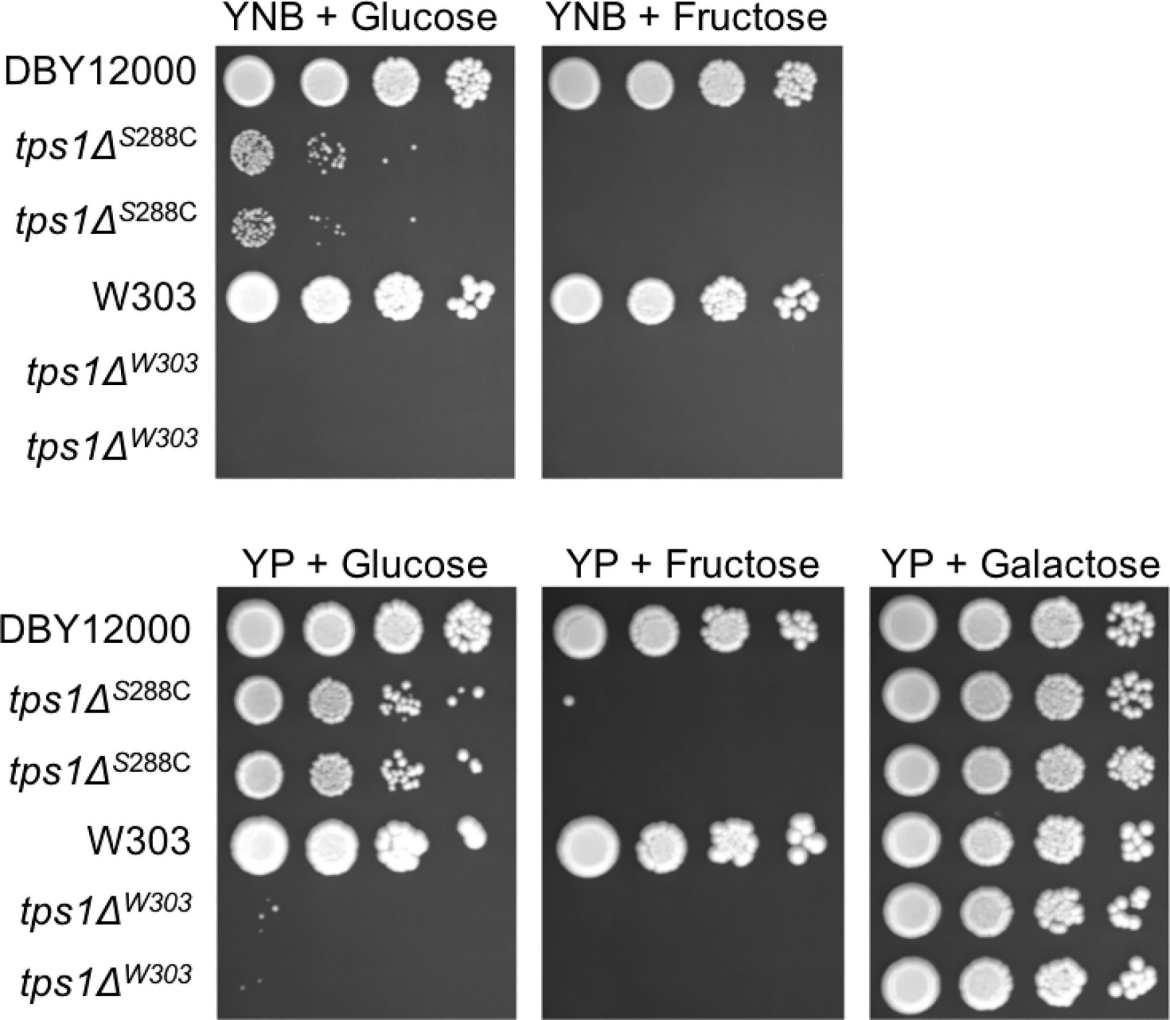
*tps1Δ* persister-like state occurs in S288C-derived strains, doesn’t occur in fructose, and is enhanced by rich media. The indicated strains were grown overnight in YNB + 2% galactose liquid before 10-fold serial dilutions were prepared and spotted onto the indicated media. Listed carbon sources were present at 2%. The initial dilution had an OD_600_ of 1.0. Plates were incubated at 30°C for 3 days. Two biological replicates of each *tps1Δ* mutant are shown (immediately below their isogenic wild type strain). Strains used in this figure: DBY12000, DBY12383, DBY15117, DBY15121.

Deletion of *TPS1*, which encodes trehalose-6-phosphate synthase, results in the inability to grow on fermentable carbon sources such as glucose or fructose [11–19]. This *tps1Δ* growth defect can be genetically suppressed by certain mutations, including deletion of the primary fermentative hexokinase gene, *HXK2* [23]. Recent work by van Heerden *et al*. demonstrated that a small fraction of *tps1Δ* cells (roughly 1 in 1000) are able to grow on glucose [13]. This is not due to a stable genetic change, as ability to grow on glucose remained at roughly 1 in 1000 after growth and re-testing of these cells, as we confirmed (Fig 1; S1 Fig). Intriguingly, while we reliably identified persister-like cells in the DBY12000 genetic background of *S*. *cerevisiae* (an S288C-derived strain), we found none in the W303 genetic background, indicating that the ability of *tps1Δ* persister-like cells to form in the presence of glucose depends on the genetic background of the yeast strain. Moreover, even in the DBY12000 strain, no persister-like cells were found when fructose was provided as the sole carbon source. Together, these results suggest that the frequency of persister-like cells is controlled by both environmental and genetic cues.

### *tps1Δ* persister-like cell frequency is affected by media composition

While performing growth assays to observe *tps1Δ* persister-like cells, we noticed that the frequency of persister-like cells is notably higher in rich media compared to minimal media (Fig 1; S1 Fig). We therefore further characterized the role that media composition plays on this phenomenon. Interestingly, *tps1Δ* persister-like cells also occur on rich media containing sucrose as a carbon source (sucrose is a disaccharide of fructose and glucose). In sucrose, the frequency of persister-like cells is enhanced at a higher growth temperature, 37°C (S2 Fig). We observed that multiple types of peptone are able to increase persister-like cell frequency, including bacto peptone, yeast extract, and tryptone peptone (S2 Fig). In contrast, neither casamino acids nor synthetic complete (SC) amino acid mix are able to enhance persister-like cell frequency (S2 Fig). Further, this phenomenon does not appear to be related to the pH of the medium (S2 Fig). Because yeast extract, and possibly other peptones, could contain some of the modified amino acids used as yeast quorum sensing molecules, we examined all the putative *S*. *cerevisiae* quorum sensing molecules and found that they did not have any effect on the *tps1Δ* persister-like state (S3 Fig) [24]. One important difference between rich and minimal yeast growth media is the nitrogen source: rich media have abundant amino acids available as a nitrogen source, whereas most minimal media use the preferred ammonium sulfate as a nitrogen source. To examine whether nitrogen source affected *tps1Δ* persister-like cell frequency, we also prepared minimal media with an amino acid mixture as a nitrogen source. Surprisingly, amino acid nitrogen sources did not enhance *tps1Δ* persister-like cell frequency, as might be expected from the results in rich media; rather *tps1Δ* persister-like cells are less prevalent in minimal media using amino acids as a nitrogen source (S4 Fig).

### Glucose and fructose are metabolically similar in wild type and *tps1Δ*

We were surprised by the observation that this phenomenon occurs in glucose but not fructose, as both are highly preferred fermentative carbon sources, and *tps1Δ* mutants generally fail to grow on either, though in most strain backgrounds fructose inhibits growth to a greater degree [11,12,14–19,22]. The metabolism of either glucose or fructose should be highly similar, supported by the observation that cells exhibit identical growth rates in either carbon source [25]. Further, glucose and fructose enter the cell via transport by the same hexose transporters and are both phosphorylated by Hxk2p in fermentative glycolysis, though enzymatic kinetics can vary [26–29]. After phosphorylation, glucose and fructose become glucose-6-phosphate and fructose-6-phosphate, respectively, which are interconvertible by glucose-6-phosphate isomerase (Pgi1p) without using any cellular energy [30]. Despite all these similarities, *tps1Δ* persister-like cells do not occur in fructose. We therefore sought to confirm whether or not glucose and fructose are treated as similar carbon sources by yeast.

**Fig 2:**
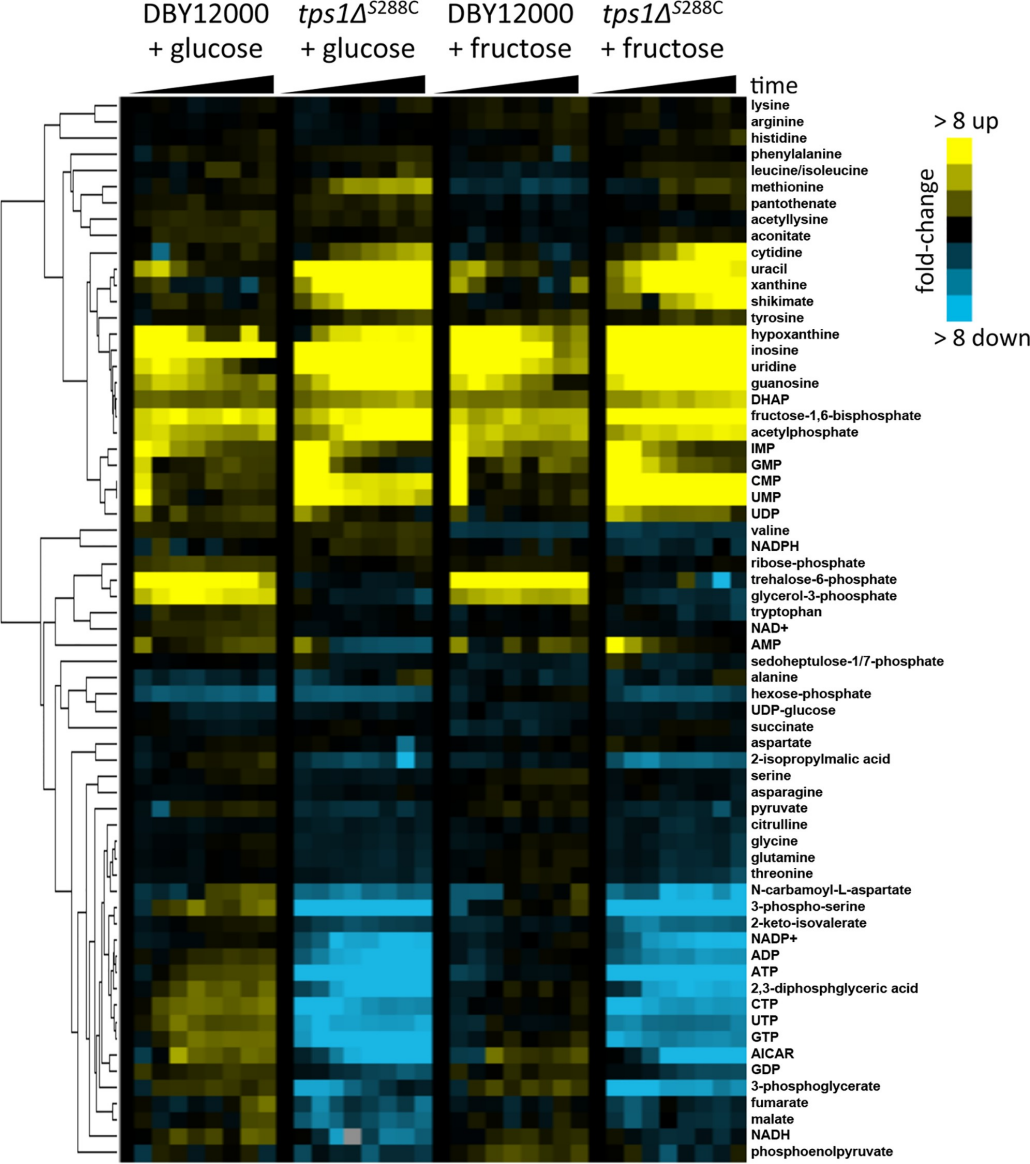
Glucose and fructose are metabolically similar in wild type and *tps1Δ*. Indicated strains were grown to mid-log phase in YNB + 2% galactose before either glucose or fructose was added to 2% (as indicated in the figure). Cells were then collected at 0, 0.5, 1, 2.5, 5, 7.5, 10, 15, and 30 minutes (indicated as time on the graph) for metabolite extraction and analysis as described in Materials and Methods. Zero-normalized metabolite changes are shown after uncentered Pearson clustering, and a legend is included to indicate relationship between metabolite fold-changes and color changes. Strains used in this figure: DBY12000, DBY12383.

First we examined metabolic changes after addition of glucose or fructose to log-phase cells grown in galactose. The metabolic response of wild type compared to *tps1Δ* is very different, with either glucose or fructose resulting in increased nucleotide degradation products and decreased metabolites associated with energy charge (Fig 2). Similar changes have been observed before, suggesting that *tps1Δ* cells are blocked at glyceraldehyde-3-phosphate dehydrogenase and are experiencing a decrease in cellular energy [13,16,20,31]. While wild type cells respond differently than *tps1Δ* cells, the wild type response to either glucose or fructose is nearly identical (Fig 2). Further, the *tps1Δ* metabolic response to either glucose or fructose is also nearly identical (Fig 2). This suggests that these two carbon sources are treated similarly from a metabolic perspective, though it is worth noting that metabolite levels alone can be poor indicators of metabolic flux.

To further investigate potential differences in how yeast cells respond to presentation of glucose or fructose, we examined gene expression responses after addition of glucose or fructose to log-phase cells grown in galactose. In contrast to the metabolic response, the gene expression responses of wild type galactose-grown cells to either glucose or fructose include a number of differences. While the overall patterns of genes that increase or decrease in expression are similar between glucose and fructose, cell cycle and transcription factor/gene expression processes are more highly expressed in glucose, while transport and membrane processes are higher in fructose (S5 Fig). This may not be surprising as glucose is sensed in multiple ways, both outside and inside the cell, to activate signaling cascades [32]. This result suggests that phenotypic differences between fructose- and glucose-grown cells likely result from differences in cellular signaling instead of metabolism per se. We also observed that the gene expression response of *tps1Δ* is very different than wild type (S5 Fig). Notably, mitochondrial functions are highly upregulated in *tps1Δ*, but not in wild type. Together, these results suggest that the *tps1Δ* persister-like state is connected with glucose sensing and signaling.

### The *tps1Δ* persister-like state is not due to intracellular phosphate depletion

**Fig 3:**
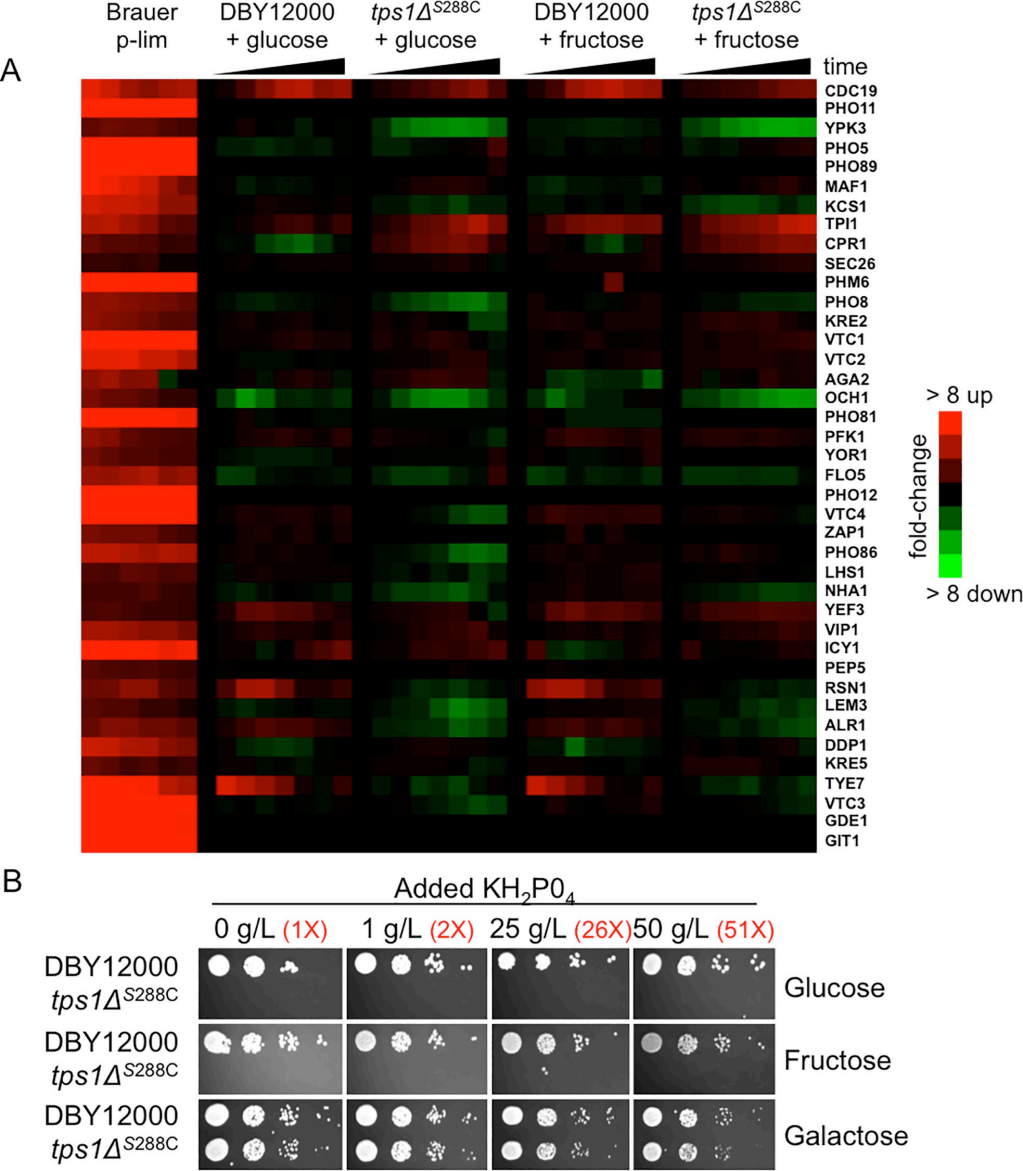
The *tps1Δ* persister-like state is not regulated by intracellular phosphate depletion. (A) Phosphate-responsive gene expression response of wild type (DBY12000) and *tps1Δ* cells grown to mid-log phase in YNB + 2% galactose before being treated with glucose or fructose (to 2%) as indicated (each column represents a time-course point: 0, 2.5, 5, 10, 15, 30, 60, and 120 minutes). Cells were collected for RNA extraction and RNA-seq analysis as described in Materials and Methods. Shown on the left for comparison are the data for genes activated by phosphate limitation from Brauer *et al*. (data is ordered as in Brauer *et al*., where gene expression from left-to-right corresponds to changing steady-state growth rates from slow to fast: 0.05, 0.1, 0.15, 0.2, 0.25, 0.3 per hour)[33]. (B) The indicated strains were grown overnight in YNB + 2% galactose liquid before 10-fold serial dilutions were prepared and spotted onto the indicated media. Phosphate was added at the indicated concentrations, with fold-change comparison to standard minimal media indicated in red. Listed carbon sources were present at 2%. The initial dilution had an OD_600_ of 0.1. Plates were incubated at 30°C for 3 days. Strains used in this figure: DBY12000, DBY12383.

One hypothesis explaining the failure of *tps1Δ* to grow on glucose is that this mutant is unable to release phosphate from trehalose-6-phosphate, thus GAPDH is unable to convert glyceraldehyde-3-phosphate into 1,3-bisphosphoglycerate in glycolysis [13,16,20,31]. We examined further the possibility that the growth defect of *tps1Δ* on fermentable carbon sources results from inorganic phosphate depletion. Previous work identified the set of genes that are activated in yeast in response to phosphate limitation, largely driven by the Pho4p transcription factor, and which includes a number of phosphate transporters and phosphatases [33,34]. We examined whether or not this set of genes was activated when *tps1Δ* grown in galactose are provided either glucose or fructose. As we show in Fig 3A, the phosphate-responsive gene set is not activated in these cells. Further, addition of phosphate to the media up to 51 times the amount present in typical minimal media does not improve *tps1Δ* growth on glucose or fructose (Fig 3B). This result is also in-line with the previously noted observation that addition of excess phosphate up to 50 mM did not suppress *tps1Δ* on glucose medium [21].

Beyond these data, the phosphate recycling hypothesis also fails to explain why deletion of *TPS2*, which accumulates trehalose-6-phosphate (and is therefore also unable to release inorganic phosphate) does not fail to grow on glucose or fructose as seen in *tps1Δ* (S6 Fig) [11,13,20,35,36]. Notably, *tps2Δ* grows poorly on galactose, especially at 37°C, and this is also not suppressed by addition of exogenous phosphate (S6 Fig). Together these results suggest that the phosphate recycling hypothesis does not explain the generation of persister-like cells, and is also not likely the main role for the trehalose metabolic pathway in glycolytic regulation.

### The *tps1Δ* persister-like state is related to respiratory complex III and cytochrome C function

**Fig 4:**
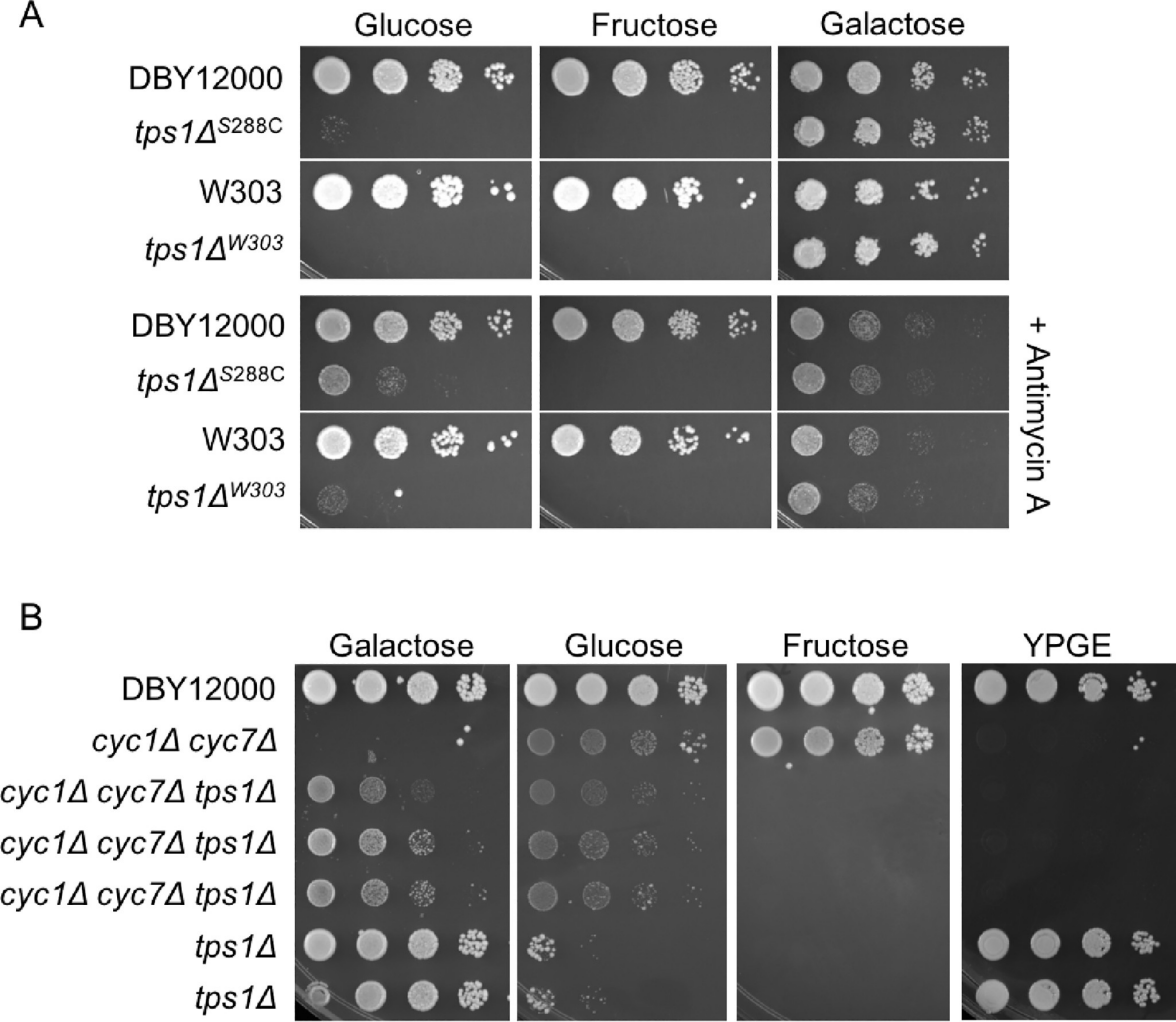
The *tps1Δ* persister-like state is enhanced by antimycin A or cytochrome C deletion. (A) The indicated strains were grown overnight in YNB + 2% galactose liquid before 10-fold serial dilutions were prepared and spotted onto the indicated minimal media. Listed carbon sources were present at 2%. Plates were incubated at 30°C for 2 days. Antimycin A was added to a final concentration of 2 μg/mL. The initial dilution had an OD_600_ of 1.0. (B) Strains were treated as described in panel A, except overnight cultures were grown in YP + 2% galactose before being spotted onto minimal media as indicated. Multiple biological replicates are included for indicated strains, and a YPGE plate incubated for 3 days at 30°C is included to demonstrate respiratory incompetence. Strains used in this figure: DBY12000, DBY12383, DBY15117, DBY15121, PGY53, PGY55, DBY12134.

In previous reports, *tps1Δ* failure to grow in glucose could be suppressed by inhibition of respiratory complex III by addition of antimycin A, myxothiazol, diuron, or deletion of *QCR9*—subunit 9 of complex III [15,17,37]. Puzzlingly, suppression of *tps1Δ* failure to grow on glucose does not occur for inhibition of complex IV, nor does it occur in petite cells [37]. We were interested in determining whether this observation was related to the *tps1Δ* persister-like state. We were able to recapitulate antimycin A suppression of the *tps1Δ* glucose growth defect in our strain background, and show that it enhances the frequency of *tps1Δ* persister-like cells (Fig 4A). Similar to the *tps1Δ* persister-like state, antimycin A has no effect on the *tps1Δ* fructose growth defect. Electrons from complex III are transferred to cytochrome C, so we also wanted to test whether disruption of cytochrome C could affect the *tps1Δ* persister-like state. To achieve complete loss of cytochrome C activity, we deleted both the *CYC1* and *CYC7* isoforms, resulting in respiratory incompetence (Fig 4B). While *cyc1Δ cyc7Δ tps1Δ* colonies are very small on minimal glucose medium, it is clear that *cyc1Δ cyc7Δ* significantly enhances formation of *tps1Δ* persister-like cells (Fig 4B). As with antimycin A, disruption of cytochrome C does not affect *tps1Δ* growth on fructose (Fig 4B). These results suggest that the cellular perturbation resulting from inhibition of either complex III or cytochrome C is related to the *tps1Δ* persister-like state, and both are likely involved with glucose sensing and signaling. While likely unrelated to the persister-like state, we also observed that *tps1Δ* is able to suppress the *cyc1Δ cyc7Δ* growth defect on minimal galactose medium, though it is not clear why this occurs (Fig 4B).

### The *tps1Δ* persister-like state is a consequence of allelic variation in *MKT1*

Despite *tps1Δ* persister-like cells not resulting from a genetic change, it is clear that there is a genetic basis behind the capacity for this phenomenon as it is largely absent in the W303 genetic background. To examine the genetic underpinnings of the capacity to exhibit *tps1Δ* persister-like cells, we mated W303- and S288C-derived *tps1Δ* strains, dissected their meiotic progeny, and scored their ability to produce *tps1Δ* persister-like cells. Between two biological replicates, the capacity to produce *tps1Δ* persister-like cells segregated roughly 2:2 among *tps1Δ* strains (29 out of 64 spores) (S7 Fig). Suppressing and non-suppressing strains were pooled together independently for each biological replicate, and genomic DNA from each pool was sequenced. Analysis of the sequencing data revealed a region on chromosome XIV where all the variants in W303 vs. S288C segregated with the capacity to produce *tps1Δ* persister-like cells; in the remainder of the genome the polymorphisms segregated independently of the persister-like phenotype (S7 Fig). This region comprises roughly 40 kilobases of DNA, and contains a number of non-synonymous mutations in genes (S8 Fig).

**Fig 5:**
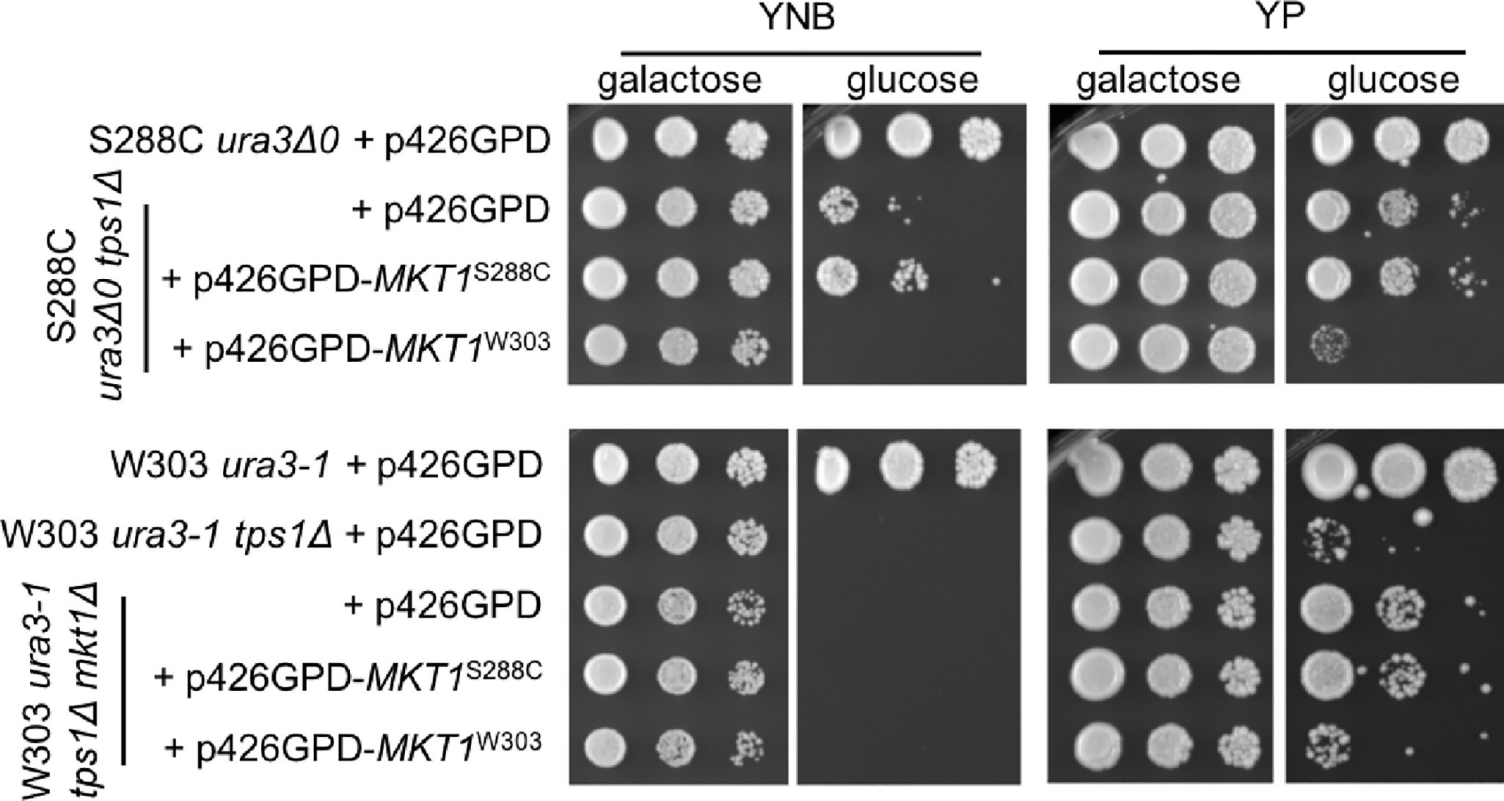
Generation of *tps1Δ* persister-like cells is a consequence of *MKT1* allelic state. Indicated strains were grown overnight in YNB + 2% galactose liquid before 10-fold serial dilutions were prepared and spotted onto the indicated media. The initial dilution had an OD_600_ of 1.0. Listed carbon sources were present at 2%. Plates were incubated at 30°C for 3 days. Strains used in this figure: DBY12045, DBY12433, DBY15039, PGY185, PGY363.

Among the list of 16 genes, a number stood out as being possible candidates due to their connections with mitochondrial function and metabolism: *MKS1*, which encodes a transcriptional regulator involved in nitrogen metabolism and mitochondria-to-nuclear signaling; *SAL1*, which encodes a mitochondrial ADP/ATP transporter; and *MKT1*, which encodes a nuclease-like protein with a multitude of described roles including regulation of mitochondrial genome stability, among others [38–42]. Swapping the W303 alleles of *MKS1* or *SAL1* into the S288C background did not prevent *tps1Δ* persister-like cells from arising (S9 Fig). In contrast, after replacing the S288C allele of *MKT1* with the W303 allele, all *tps1Δ* strains containing the W303 allele of *MKT1* failed to generate *tps1Δ* persister-like cells at normal S288C levels (S10 Fig). To confirm the causality of *MKT1* alleles in regulating the *tps1Δ* persister-like state, we expressed both the S288C or W303 alleles in an S288C-derived strain. Overexpression of the S288C *MKT1* allele had a similar level of *tps1Δ* persister-like cells compared to an empty vector control (Fig 5). In contrast, overexpression of the W303 allele prevented *tps1Δ* persister-like formation in minimal media and greatly inhibited formation in rich media (Fig 5). The latter experiment was performed in a strain containing the native S288C *MKT1* allele, suggesting that activation of *tps1Δ* persister-like state by this allele is recessive to the W303 allele. To further test this, we observed that overexpression of the S288C *MKT1* allele in a W303 *tps1Δ* strain containing its genomic copy of the *MKT1* allele does not exhibit *tps1Δ* persister-like cells, again suggesting that the S288C *MKT1* allele is recessive with regard to this phenotype (S11 Fig). Finally, we wanted to test whether or not disruption of *MKT1*, or expression of the S288C *MKT1* allele, could induce *tps1Δ* persister-like cells in the W303 genetic background. Both deletion of *MKT1* and expression of the S288C *MKT1* allele greatly increased *tps1Δ* persister-like formation on rich media, though not on minimal medium (Fig 5). Further, expression of the W303 *MKT1* allele returned persister-like formation to the same low level observed in a strain containing its genomic copy of *MKT1*. This suggests that the S288C *MKT1* allele is not only recessive to the W303 allele, but is a loss-of-function allele with regard to this phenotype, as also suggested by Swinnen *et al*. [43]. Taken together, these results suggest that allelic variation in *MKT1* regulates the ability to enter the *tps1Δ* persister-like state.

## Discussion

In this paper, we recapitulate the observations of van Heerden *et al*. that a small fraction of cells in galactose-grown cultures of *tps1Δ* are able to grow in the presence of glucose, and that this is not due to genetic suppression [13]. We then extend these observations in a number of ways, including characterizing the dependency of this effect on various nutrient sources and functional complex III or cytochrome c in the electron transport chain. van Heerden *et al*. observed the same phenomenon and concluded that the trehalose biosynthetic pathway plays an essential role in regulating glycolysis, functioning as a futile cycle to slow upper glycolysis, while providing inorganic phosphate to GAPDH in lower glycolysis [13,16,20]. This “phosphate recycling” theory has been suggested a number of times, and is also supported by the observations that glucose-treated *tps1Δ* cells rapidly deplete all cellular polyphosphate stores, the *tps1Δ* mutant is blocked at the phosphate-requiring GAPDH enzyme when exposed to fermentable carbon sources, and that *tps1Δ* can be suppressed by activation of glycerol production, which liberates inorganic phosphate [13,16,20,44]. However, this model for the role of trehalose metabolism in glycolytic regulation is not supported based on the evidence provide here. Addition of excess phosphate does not reverse this phenotype, which has also been described previously; nor are the phosphate-starvation genes activated [21]. Additionally, the *tps2Δ* mutant is also unable to liberate inorganic phosphate from trehalose-6-phosphate, and has long been known as able to grow on both glucose and fructose [35,36]. It is also worth noting that the defect associated with *tps1Δ* cannot be strictly defined by loss of a glycolytic shunt to slow down overactive glycolysis, because glucose-6-phosphate is still siphoned from glycolysis to increased glycogen in *tps1Δ* [45,46]. Further work is needed to better understand the molecular connections between trehalose metabolism and regulating fermentation.

One of the striking observations related to these persister-like cells is that they only occur in glucose and not fructose, despite both being highly preferred, fermentable carbon sources. It has been long known that *tps1Δ* cells exhibit more extreme growth inhibition in fructose compared to glucose, though the precise mechanism remains unclear [11,12,14–19,22]. Interestingly, in *tps1*^-^ mutants the genetic suppression rate in glucose appears to be higher than observed for fructose, though it isn’t clear how the mechanisms associated with genetic suppression are related to the mechanism of persistence, if at all [47]. From an energetic perspective, both glucose and fructose are similar distances from fructose-1,6-bisphosphate (FBP): both are transported by Hxt proteins, and both are phosphorylated by Hxk proteins. Once phosphorylated, glucose-6-phosphate and fructose-6-phosphate are interchangeable by phosphoglucoisomerase, an enzymatic reaction that requires no energy input. In a typical cell with active glycolysis, unidirectional phosphorylation of F6P to FBP will also drive the phosphoglucoisomerase reaction in the direction of F6P. In-line with their similar catabolic path, our metabolomics data suggested that these carbon sources are generally equivalent based on how they alter concentrations of other metabolites (Fig 2). Though notably over the 30 minute metabolomics time course, we did observe that in fructose vs. glucose, FBP accumulates more quickly and 3-phosphoglycerate depletes more quickly, both suggesting that fructose exposed cells are experiencing glycolytic dysregulation slightly faster. In contrast, our gene expression data highlights some differences between the two sugars, mostly related to transcription (signaling, chromatin organization, RNA polymerase II activity, etc.). This is likely due to the fact that there are multiple glucose-specific sensors associated with yeast cells, including Gpr1 [48,49]. These results suggest that physiological differences between these glucose and fructose may be more related to regulatory effects rather than direct metabolic effects. It is also possible that regulation of which hexokinase and hexose transport enzymes are functionally expressed could be related to differential responses between fructose and glucose.

We also observed that the *tps1Δ* persister-like state rarely occurs in the W303 genetic background, but robustly in our S288C-derived genetic background, which opened the door for genetic analysis of this trait. The genetic capacity for generating *tps1Δ* persister-like cells is linked to the S288C-specific *MKT1* allele (G30D and R453K relative to a consensus “wild type” observed in W303, RM11a, and a variety of other *Saccharomyces* strains)[41]. While the molecular role of Mkt1 remains unknown, the protein contains multiple nuclease-like domains and deletion of the gene has a variety of reported phenotypes: maintenance of K2 killer toxin, post-translational regulation of HO endonuclease, post-transcriptional regulation of stress-induced genes, and regulation of mitochondrial genome stability [38,39,41,42]. Observation of Mkt1 co-localization in P-bodies after ethanol stress also supports a role in post-transcriptional regulation of gene expression [42]. In addition, *MKT1* polymorphisms appear in quantitative trait mapping studies as responsible, at least in part, for variations in doubling time in multiple carbon sources, sporulation, high temperature growth, and resistance to ethanol or a number of toxic compounds [40–43,50–55]. Interestingly, the S288C allele of *MKT1* generally segregates with stress sensitivity in crosses with the RM11a background, with decreased sporulation rate in a cross with the SK1 background [40–42,50–55], and with lower ethanol tolerance in a cross with bioethanol production strain VR1. However, in both our S288C and W303 backgrounds, deletion or allele-swapping *MKT1* does not appear to confer any general issues in growth or survival using standard laboratory conditions (Fig 5). It is clear that elucidating the detailed cellular role of Mkt1 will have a significant impact in our understanding of a variety of processes.

An interesting possible mechanism to explain persister-like cell formation is based on a recent paper demonstrating that *tps1Δ* cells exposed to glucose hyperaccumulate FBP, which hyperactivates Ras, triggering apoptosis [56,57]. Based on this model, it is possible that some *tps1Δ* cells escape apoptosis if they exhibit a slower accumulation of FBP, though at this time there are no analytical methods for tracking FBP levels in single, living cells over time. Our metabolomics data was performed at the population level, and by 30 minutes we observed similar increases in FBP and similar decreases in ATP in both glucose and fructose (Fig 2). However, accumulation of FBP did appear to have slower kinetics after glucose addition when compared to fructose. Another connection between the Ras pathway and persister-like cell formation is based on the failure of fructose to produce persister-like cells. Ras is activated by the Gpr1 protein, which acts as a glucose sensor but does not detect fructose [48,49]. Further, it is interesting that we observed deletion of cytochrome c can also increase persister-like cell formation, as cytochrome c is known for having an role independent of the electron transport chain: activating apoptosis in cells with stressed/damaged mitochondria [58]. Future studies exploring the connection between persister-like cells, electron transport chain, apoptosis, and the Ras pathway may lead to a better understanding of the mechanisms controlling this phenomenon. Additionally, while escaping Ras-mediated apoptosis provides a potential mechanism, it does not explain the role of *MKT1* in persister-like cell formation.

We conclude that the *tps1Δ* persister-like state appears to be a complex interaction between the nutrient environment and mutations present in the S288C genetic background that affect metabolic control and glucose sensing/signaling. Many aspects of this phenomenon remain unclear, including why disabling Complex III or cytochrome c of the electron transport chain increases generation of persister-like cells, why there is such a significant difference between glucose and fructose, how generation of persister-like cells is enhanced by addition of peptone, or why *tps1Δ* persister cells only occur in minimal media with ammonium as a nitrogen source. More work will be required to elucidate the connections between these pathways and develop a model for the role of trehalose metabolism sufficient to comprehensively explain the many known growth and metabolic phenotypes. Finally, identifying a genetic regulator of persister-like cell formation in yeast suggests that persister phenotypes in other eukaryotic cells, such as cancer cells, may also be genetically regulated, as already observed in bacteria [59–61].

## Materials and methods

### Yeast media

Yeast cell growth and standard laboratory manipulations were performed as described [62]. All media used was either minimal medium (0.67% Yeast Nitrogen Base without amino acids plus 2% indicated carbon sources), or rich medium (2% bacto peptone, 1% yeast extract, 2% indicated carbon sources). Exceptions are noted in the text (for example, YPGE medium contained 2% bacto peptone, 1% yeast extract, 3% glycerol, and 2% ethanol).

### Yeast growth

Measurements of cell density were also performed by measuring absorbance at 600 nm using a Genesys 6 spectrophotometer (Thermo Scientific). Culture growth was also measured using a Synergy H1 Hybrid reader (BioTek) with 200 μL cultures in a 96-well plate (plate was sealed with a Breathe-Easy gas-permeable membrane from Research Products International Corporation). For comparative growth assays, cells were spotted onto relevant media. This involved dilution of a culture to an OD_600_ of either 0.1 or 1.0, as described in accompanying figure legends, followed by 10-fold serial dilutions. All dilutions were then spotted onto solid media using a Replica Plater for 96-well Plate, 8x6 array (Sigma-Aldrich). For each comparative growth assay figure, at least three biological replicates were performed, and a representative example is shown.

### Yeast strain and plasmid construction

All strains were made in the DBY12000 S288C-derived background, or the W303 genetic background (Table 1). Gene deletions were made by transformation into a diploid to produce a heterozygote, which was confirmed by PCR, then dissected to get MAT**a** and MATα segregants. All combinatorial gene deletion/insertion strains were made by mating, sporulating, and tetrad dissection. Sporulation was performed by growing cells to log phase in rich media, collecting cells by centrifugation, washing once in 1% potassium acetate, then resuspending in 1% potassium acetate. Cells were then incubated at room temperature on a roller wheel for at least 4 days before tetrad dissection. Allele swaps were done using the *delitto perfetto* method as described [63]. To construct *MKT1* expression plasmids (Table 2), linearized p426GPD (using SpeI and XhoI) was transformed into *ura3*^-^ yeast cells along with *MKT1* PCR products containing 40 b.p. of flanking sequence identical to the 3’ end of the *TDH3* promoter or to the 5’ end of the *CYC1* 3’UTR present in p426GPD. Plasmids were assembled by homologous recombination in yeast, then were extracted using Zymoprep Yeast Plasmid Miniprep II kit (Zymo Research). Extracted plasmids were transformed into TOP10 *E*. *coli* for storage and amplification. Gene insertion and allele-specific mutations were confirmed by Sanger sequencing.

**Table 1:** Strains used in this study.

Strain	Genotype	Reference
DBY12000	S288C MAT**a** prototrophic *HAP1*^+^ derivative of FY4	see below^a^
DBY12001	S288C MATα prototrophic *HAP1*^+^ derivative of FY4	see below^a^
DBY12045	S288C MAT**a** *ura3Δ0*	this work
DBY12118	S288C MAT**a** *tps2Δ*::natAC	this work
DBY12134	S288C MAT**a** *tps1Δ*::kanMX	this work
DBY12383	S288C MAT**a** *tps1Δ*::natAC	[9]
DBY12433	S288C MAT**a** *tps1Δ*::natAC *ura3Δ0*	this work
DBY12688	S288C/W303 *TPS1*/*tps1Δ*::kanMX (DBY12134 x DBY15118)	this work
DBY12689	S288C/W303 *TPS1*/*tps1Δ*::kanMX (DBY12001 x DBY15121)	this work
DBY12795	S288C *TPS1*/*tps1Δ*::natAC *SAL1*/*sal1Δ*::*SAL1*^W303^ *URA3/ura3Δ0*	this work
DBY12796	S288C *TPS1*/*tps1Δ*::natAC *MKS1*/*mksΔ*::*MKS1*^W303^ *URA3/ura3Δ0*	this work
DBY12813	S288C MAT**a** *tps2Δ*::kanMX	this work
DBY12821	S288C *TPS1*/*tps1Δ*::natAC *MKT1*/*mktΔ*::*MKT1*^W303^ *URA3/ura3Δ0*	this work
DBY15039	W303 MATα *ura3-1*	this work^c^
DBY15117	W303 MAT**a** prototroph	this work^c^
DBY15118	W303 MATα prototroph	this work^c^
DBY15121	W303 MAT**a** *tps1Δ*::kanMX	this work
PGY53	S288C MATα *cyc1Δ*::kanMX *cyc7Δ*::hphMX	this work
PGY55	S288C MAT**a** *cyc1Δ*::kanMX *cyc7Δ*::hphMX *tps1Δ*::natAC	this work
PGY185	W303 MAT**a** *tps1Δ*::kanMX *ura3-1*	this work
PGY363	W303 MAT**a** *tps1Δ*::kanMX *ura3-1 mkt1Δ*::natAC	this work

^a^This strain is a GAL^+^ prototrophic derivative of S288C. The details for construction of this strain are found in Hickman and Winston [64], while the first article using this strain is Hickman *et al* [65]. The strain was a kind gift from the Winston lab (where it is named FY2648). All S288C-derived strains listed above contain the repaired *HAP1* allele.

^b^natAC refers to a version of the natMX dominant drug resistance marker cassette that contains a yeast codon-optimized nat^r^ gene. This cassette was a kind gift from Amy Caudy.

^c^These strains were generated by crossing W303 derivatives that were kind gifts from Fred Cross (the original prototrophic MAT**a** W303 strain from Fred Cross is called 2832-1B).

**Table 2:** Plasmids used in this study.

Strain	Plasmid	Reference
RB3622	p426GPD (2μ, *URA3*^+^, amp^r^)	[66,67]
PGB27	p426GPD-MKT1-S288C (2μ, *URA3*^+^, amp^r^)	this work
PGB28	p426GPD-MKT1-W303 (2μ, *URA3*^+^, amp^r^)	this work

### RNA extraction, RNA-seq, and gene expression analysis

For gene expression analysis, samples were collected for RNA extraction by vacuum filtration onto nylon filters. Cells were grown in batch culture in 100 mL of YNB + 2% galactose in a 500 mL baffled flask to an OD600 between 0.230 and 0.273, then after addition of glucose or fructose to 2%, samples were collected at 2.5, 5, 10, 15, 30, 60, and 120 minutes. Filters were immediately placed into tubes, submerged into liquid nitrogen and stored at -80°C until RNA extraction. RNA was extracted by the acid-phenol method and cleaned using RNeasy mini columns (Qiagen). RNA-seq libraries were prepared using poly-dT selection of polyA transcripts in combination with the Illumina Tru-seq stranded mRNA preparation kit (Illumina). Production of cDNA in this step was performed using SuperScriptIV (Invitrogen). cDNA fragments ranging from 200–600 b.p. were selected using a Pippin-HT (Sage Science). Sequencing was performed on an Illumina HiSeq 4500 (Illumina). Basecalls were performed using CASAVA version 1.4 (Illumina). Sequencing reads were aligned to the SacCer3 assembly and the Saccharomyces Genome Database transcriptome using TopHat2 with the following options: -p 4,—library-type fr-firststrand, and -M [68–70]. Transcript abundances were estimated using Cufflinks CuffNorm with default options [68]. Before zero-normalization and log2-conversion, all FPKM values below 10 were floored to 10 to avoid artificially inflated fold changes associated with low-abundance genes. Data was then filtered to analyze only known protein-coding genes (primarily removed dubious ORFs and retrotransposon elements). All RNA-seq data was deposited with the NCBI Gene Expression Omnibus (GEO reference number GSE103285). Each time-series experiment was zero-normalized before hierarchical clustering was performed (Pearson uncentered metric, average linkage) using Cluster 3.0 (http://bonsai.hgc.jp/~mdehoon/software/cluster/software.htm). Data was visually represented, examined, and exported using Java TreeView version 1.1.6r2 [71]. Clusters were selected visually, and genes in each cluster were examined for functional enrichment using GO Slim search tools available on SGD (http://www.yeastgenome.org/cgi-bin/GO/goSlimMapper.pl).

For comparison to genes that have increased expression in response to phosphate limitations, the entire data set from Brauer *et al*. was downloaded from http://growthrate.princeton.edu/ [33]. Hierarchical clustering was performed (Pearson uncentered metric, average linkage) using Cluster 3.0, and the phosphate-limitation-specific cluster was selected and exported using Java TreeView version 1.1.6r2. Our gene expression data was then mapped onto that set of genes. Expression data from Brauer et al. for phosphate-limitation-induced genes is also shown to the left of gene expression data generated here (data is ordered as in Brauer *et al*., where gene expression from left-to-right corresponds to changing steady-state growth rates from slow to fast: 0.05, 0.1, 0.15, 0.2, 0.25, 0.3 per hour).

### Metabolite profiling

For metabolomic profiling, roughly 7.5 x 10^7^ cells were collected at each time-point (0, 0.5, 1, 2.5, 5, 7.5, 10, 15, and 30 minutes), filtered onto a nylon membrane, and quenched in an 80:20 mixture of HPLC-grade methanol and HPLC-grade water at -20°C. This was allowed to chill at -20°C for at least 20 minutes before the cell material slurry was repeatedly pipetted over the filter and collected into a microcentrifuge tube. The extraction solvent/cell slurry was centrifuged at 4°C for 5 minutes, and a fraction of this supernatant was dried under nitrogen gas for resuspension and metabolite profiling using liquid chromatography coupled to mass spectrometry (LC-MS). Each sample was examined using two different analytical separation methods. One method included reverse-phase ion-pairing LC on an Agilent Extend C18 column with tributylamine as an ion-pairing agent, followed by metabolite identification using a Q Exactive Plus mass spectrometer (Thermo Scientific). The other method included LC on a ZIC pHILIC column (SeQuant) followed by metabolite identification using a Q Exactive Plus mass spectrometer (Thermo Scientific)[72–74]. Data were then analyzed using the open-source software Metabolomic Analysis and Visualization ENgine (MAVEN)[75].

## Supporting information

S1 File Metabolomics data. This tab-delimited text file contains the metabolomics data used to produce the heat map in Fig 2. The first row indicates carbon source, strain, and time-point as described in Materials and Methods (0, 0.5, 1, 2.5, 5, 7.5, 10, 15, and 30 minutes). Each data value represents the peak height for each metabolite listed in the first column.
(TXT)

S2 File Phosphate-limitation RNA-Seq data. This tab-delimited text file contains the normalized RNA-Seq data used to produce the heat map in Fig 3A. The columns labeled “Brauer” include the chemostat growth rate in the column label. Remaining columns are labeled with the strain, carbon source switch, and time-point as described in Materials and Methods (0, 2.5, 5, 10, 15, 30, 60, 120 minutes). Data values for Brauer columns were downloaded as described in Materials and Methods. Remaining data cells include log_2_-normalized mRNA levels relative to the time-zero (zero-normalized for each time-course).
(TXT)

S3 File All RNA-Seq data. This tab-delimited text file contains the processed RNA-Seq data used to produce the heat map in Fig 3A and S5 Fig (processing is described in Materials and Methods). Column labels include the strain, carbon source switch, and time-point as described in Materials and Methods (0, 2.5, 5, 10, 15, 30, 60, 120 minutes). Data values are log_2_-normalized mRNA levels relative to the time-zero (zero-normalized for each time-course).
(TXT)

S1 Fig The *tps1Δ* persister-like state is non-genetic and is enhanced by rich media. **A**. *tps1Δ* cells were grown overnight in YNB + 2% galactose, then 1:10 serial dilutions were spread onto the indicated media. Plates were incubated for 2–3 days at 30°C before photographing. **B**. A single colony from the plate indicated by red outline in panel A was re-grown in YNB + 2% galactose, then treated as described for panel A. **C**. A single colony from the plate indicated by green outline in panel A was re-grown in YNB + 2% galactose, then treated as described for panel A. Strain used in this figure is DBY12383.
(PDF)

S2 Fig Multiple types of peptone influence the *tps1Δ* persister-like state, and this is independent of media pH. A. Wild type (DBY12000) and *tps1Δ* cells were grown overnight in YNB + 2% galactose, then 1:10 serial dilutions were spread onto the indicated media (initial dilution OD_600_ = 1.0). Plates were incubated for 3 days at the indicated temperatures before photographing. The pH of each type of plate was measured with a pH strip and listed in parentheses. B. Similar experiment as shown in panel A, though performed on a different day with a number of different media conditions as indicated. The media pH indicated in red for YP Sucrose was adjusted to 5.5 using HCl (typical pH for minimal media without amino acid supplementation). Strains used in this figure: DBY12000, DBY12383.
(PDF)

S3 Fig Yeast quorum sensing molecules do not influence the *tps1Δ* persister-like state. The indicated strains were grown overnight in YNB + 2% galactose, then 1:10 serial dilutions were prepared (initial dilution OD_600_ = 1.0). Strains were spotted onto the indicated media. Top row: comparing two carbon sources as indicated (both present at 2%). Bottom row: all YNB + 2% glucose plates containing the indicated quorum sensing molecules at 500 μM. Two biological replicates of *tps1Δ* were included for each strain background. Notably, the W303 wild type strain appears unable to grow in the presence of 500 μM farnesol. Strains used in this figure: DBY12000, DBY12383, DBY15117, DBY15121.
(PDF)

S4 Fig The *tps1Δ* persister-like state is dependent on ammonia as a nitrogen source. **A**. The indicated strains were grown overnight in YNB + 2% galactose, then 1:10 serial dilutions were prepared (initial dilution OD_600_ = 1.0). Strains were spotted onto the indicated media containing carbon sources at 2%. Three biological replicates of *tps1Δ* were included for each strain background. **B**. The indicated strains were grown overnight in YNB + 2% galactose, then 1:10 serial dilutions were prepared (initial dilution OD_600_ = 1.0). Strains were spotted onto the indicated media containing various nitrogen sources. Usable nitrogen was present at 76 mM (the amount in typical minimal medium). 2–3 biological replicates of *tps1Δ* were included for each strain background. All plates were incubated for 3 days at 30°C before photographing. Strains used in this figure: DBY12000, DBY12383, DBY15117, DBY15121, DBY12118.
(PDF)

S5 Fig The gene expression response of wild type and *tps1Δ* cells to glucose and fructose exhibits strain- and condition-dependent effects. Strains were grown to early log phase in YNB + 2% galactose before addition of glucose or fructose to 2% as indicated. Cells were sampled at 0, 2.5, 5, 10, 15, 30, 60, and 120 minutes for RNA preparation. RNA-seq libraries and sequencing were performed as described in Materials and Methods. Data was analyzed as described in Materials and Methods. **A—**Heat- map showing wild type only. Indicated genes are *YDR524W-C (gene of unknown function) and **RPL41B (ribosomal 60S subunit L41B). **B—**Heat-map showing wild type and *tps1Δ*. In both A and B, individual clusters are indicated to the right of the heat-map, along with the Pearson correlation for each cluster shown parenthetically. **C—**Significantly enriched GO terms from highlighted clusters in panels A and B. GO terms were identified by searching using the SGD (www.yeastgenome.org) GO Slim Mapper tool, and examining Process (P), Function (F), Component (C), and Macromolecular Complex (M) data sets. Listed are terms manually curated as highly significant. Strains used in this figure: DBY12000, DBY12383.
(PDF)

S6 Fig Neither *tps1Δ* nor *tps2Δ* growth defects are restored by addition of exogenous phosphate to growth media. Strains were grown overnight in minimal media (glucose-containing for *tps2Δ*, galactose-containing for *tps1Δ*), then 1:10 serial dilutions were prepared (initial dilution OD_600_ = 1.0) and spotted onto the indicated media and incubated at indicated temperatures for 3 days before photographing. Strains used in this figure: DBY12000, DBY12134, DBY12383, DBY12118, DBY12813.
(PDF)

S7 Fig Segregation of *tps1Δ* persister-like activity to a region on chromosome XIV. Segregants from DBY12689 (**A**) and DBY12688 (**B**) were spotted onto the indicated minimal media and grown 3 days at 30°C (3 x 10-fold serial dilutions; initial dilution OD_600_ = 1.0). **C**. Strains with *tps1Δ* persister- like activity (**+**) and strains without (**-**) were pooled from both sets of segregants and sequenced. Shown is a IGV browsing window zoomed into chromosome XIV. Because the reference for sequence alignment was an S288C derivative, all W303-specific mutations appear as colored vertical bars. Note that strains that fail to exhibit *tps1Δ* persister-like activity (**-**) all contain the W303 genetic material for this region, while the opposite is true for those segregants that do exhibit *tps1Δ* persister-like activity (**+**).
(PDF)

S8 Fig Potential causative mutations on chromosome XIV between 440,000 and 490,000 kb (within coding regions). (PDF)

S9 Fig Mutations in *MKS1* or *SAL1* in S288C compared to W303 are not responsible for *tps1Δ* persister-like activity. The indicated strains, including *URA3*+ segregants from DBY12796 (*MKS1* test) and DBY12795 (*SAL1* test) were grown overnight in YNB + 2% galactose. Next, 1:10 serial dilutions were prepared (initial dilution OD_600_ = 1.0) and strains were spotted onto the indicated media, then incubated for 3 days at 30°C.
(PDF)

S10 Fig *MKT1* regulates the *tps1Δ* persister-like state: *URA3*+ *tps1Δ* segregants from DBY12821 (along with the indicated wild type and *tps1Δ* controls) were grown overnight in YNB + 2% galactose liquid before 10-fold serial dilutions were prepared and spotted onto the indicated media. The initial dilution had an OD_600_ of 1.0. Listed carbon sources were present at 2%. Plates were incubated at 30°Cfor 3 days. Cells from each strain shown were grown overnight in YP + 2% Galactose for genomic DNA preparation, *MKT1* PCR amplification, and sequencing. The identified allele present is indicated for each strain: red for the W303 allele, or blue for the S288C allele.
(PDF)

S11 Fig Overexpression of *MKT1*S288C in W303 tps1 does not induce persister-like state, suggesting the *MKT1*S288C allele is recessive. Indicated strains were grown overnight in YNB + 2% galactose liquid before 10-fold serial dilutions were prepared and spotted onto the indicated media. The initial dilution had an OD_600_ of 1.0. Listed carbon sources were present at 2%. Plates were incubated at 30°C for 3 days. Strains used in this figure: DBY15039, PGY185.
(PDF)

## Abbreviations

GAPDH: glyceraldehyde-3-phosphate dehydrogenase
