# Supplementary material for: A tps1Δ persister-like state in Saccharomyces cerevisiae is regulated by MKT1
Source: PLoS One. 2020 May 29;15(5):e0233779. doi: 10.1371/journal.pone.0233779 (PMC7259636; doi:10.1371/journal.pone.0233779)
Supplement: S1 Fig — A. tps1Δ cells were grown overnight in YNB + 2% galactose, then 1:10 serial dilutions were spread onto the indicated media. Plates were incubated for 2–3 days at 30°C before photographing. B. A single colony from the plate indicated by red outline in panel A was re-grown in YNB + 2% galactose, then treated as described for panel A. C. A single colony from the plate indicated by green outline in panel A was re-grown in YNB + 2% galactose, then treated as described for panel A. Strain used in this figure is DBY12383. (PDF) [file pone.0233779.s004.pdf]

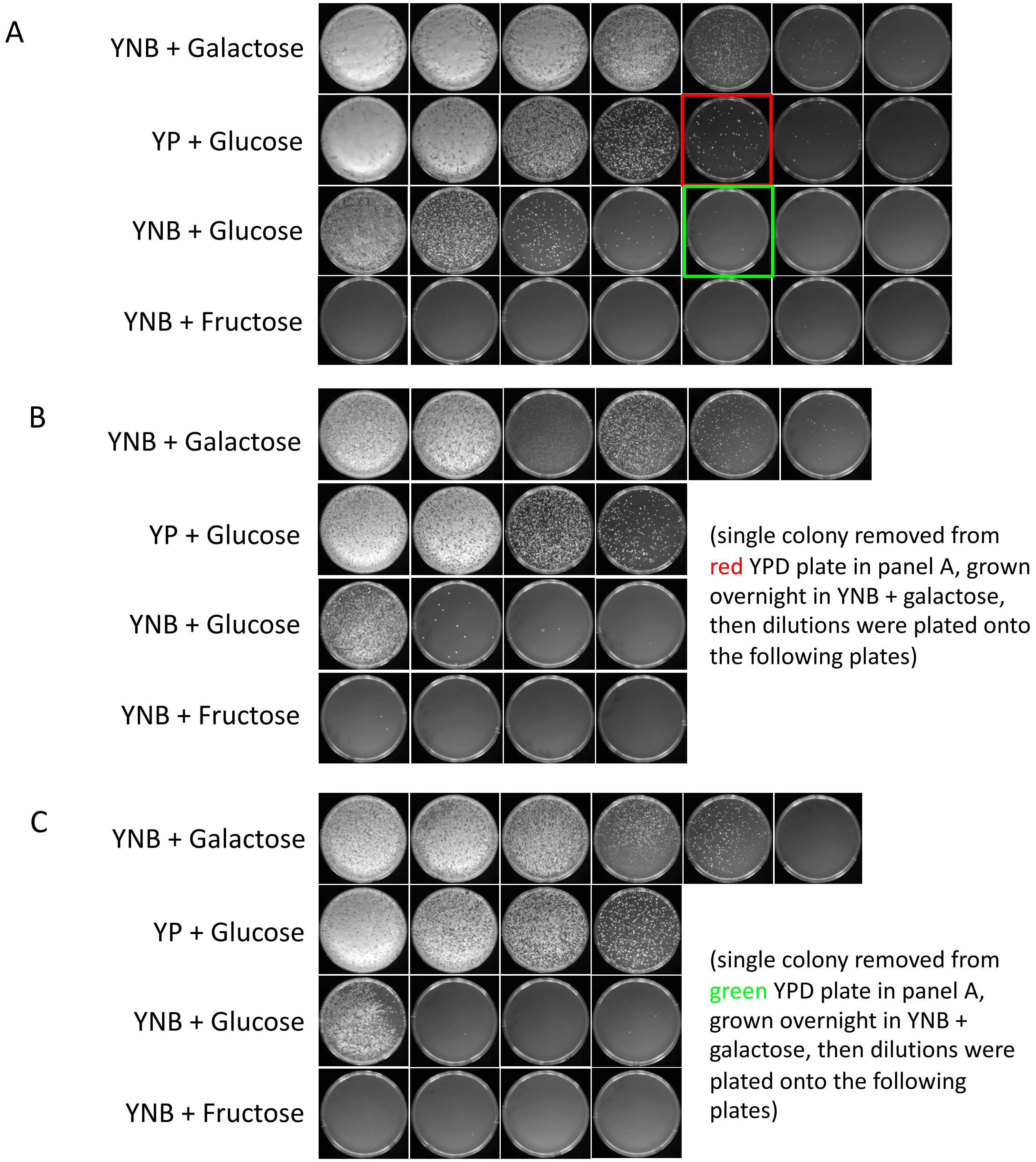

Supplemental Figure 1. The *tps1Δ* persister-like state is non-genetic and is enhanced by rich media. A. *tps1Δ* cells were grown overnight in YNB + 2% galactose, then 1:10 serial dilutions were spread onto the indicated media. Plates were incubated for 2-3 days at 30°C before photographing. **B**. A single colony from the plate indicated by red outline in panel A was re-grown in YNB + 2% galactose, then treated as described for panel A. **C**. A single colony from the plate indicated by green outline in panel A was re-grown in YNB + 2% galactose, then treated as described for panel A. Strain used in this figure is DBY12383.
