# Supplementary material for: A tps1Δ persister-like state in Saccharomyces cerevisiae is regulated by MKT1
Source: PLoS One. 2020 May 29;15(5):e0233779. doi: 10.1371/journal.pone.0233779 (PMC7259636; doi:10.1371/journal.pone.0233779)
Supplement: S2 Fig — A. Wild type (DBY12000) and tps1Δ cells were grown overnight in YNB + 2% galactose, then 1:10 serial dilutions were spread onto the indicated media (initial dilution OD600 = 1.0). Plates were incubated for 3 days at the indicated temperatures before photographing. The pH of each type of plate was measured with a pH strip and listed in parentheses. B. Similar experiment as shown in panel A, though performed on a different day with a number of different media conditions as indicated. The media pH indicated in red for YP Sucrose was adjusted to 5.5 using HCl (typical pH for minimal media without amino acid supplementation). Strains used in this figure: DBY12000, DBY12383. (PDF) [file pone.0233779.s005.pdf]

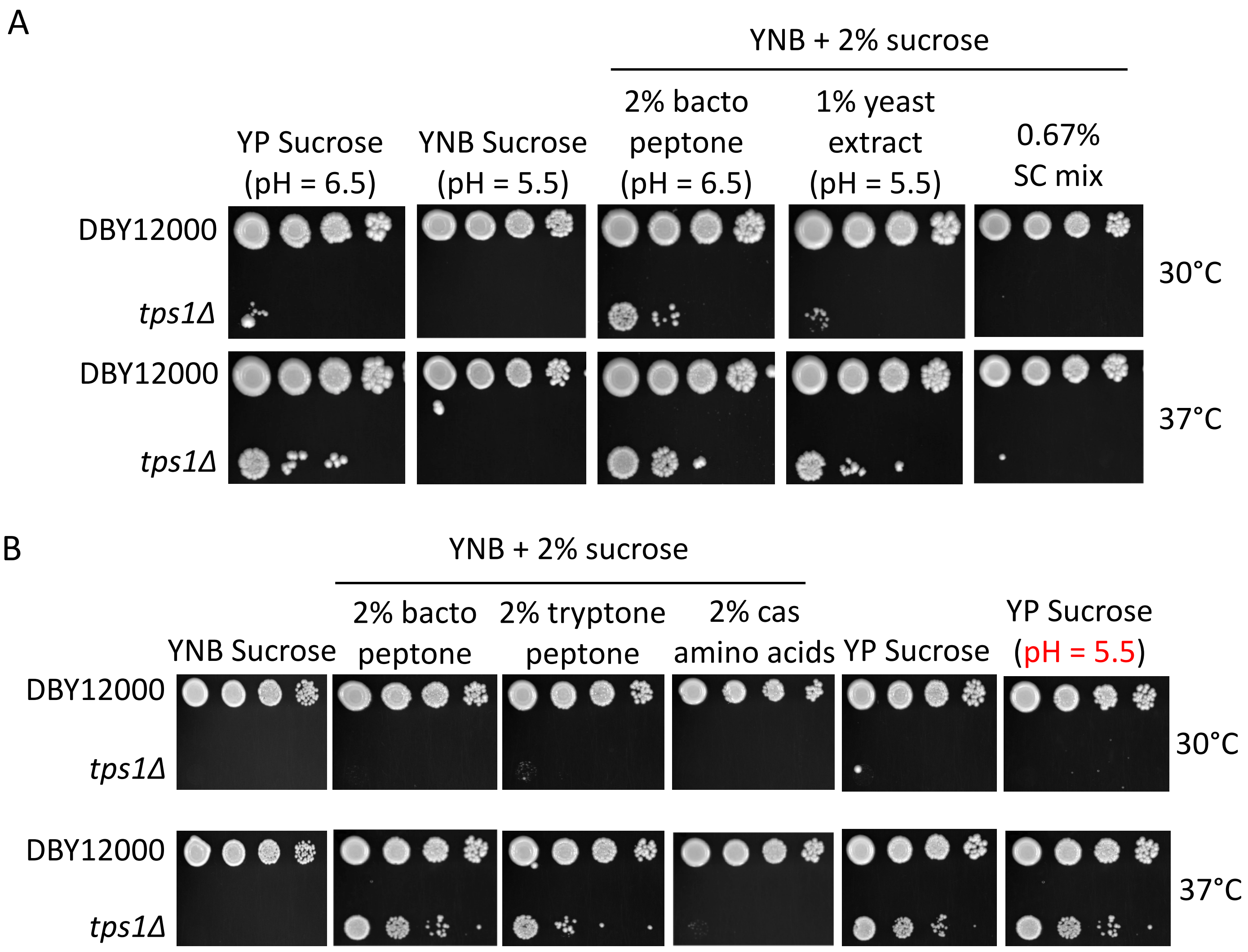

Supplemental Figure 2. Multiple types of peptone influence the *tps1Δ* persister-like state, and this is independent of media pH. **A**. Wild type (DBY12000) and *tps1Δ* cells were grown overnight in YNB + 2% galactose, then 1:10 serial dilutions were spread onto the indicated media (initial dilution OD_{600} = 1.0). Plates were incubated for 3 days at the indicated temperatures before photographing. The pH of each type of plate was measured with a pH strip and listed in parentheses. **B**. Similar experiment as shown in panel A, though performed on a different day with a number of different media conditions as indicated. The media pH indicated in red for YP Sucrose was adjusted to 5.5 using HCl (typical pH for minimal media without amino acid supplementation). Strains used in this figure: DBY12000, DBY12383.
