# Supplementary material for: A tps1Δ persister-like state in Saccharomyces cerevisiae is regulated by MKT1
Source: PLoS One. 2020 May 29;15(5):e0233779. doi: 10.1371/journal.pone.0233779 (PMC7259636; doi:10.1371/journal.pone.0233779)
Supplement: S3 Fig — The indicated strains were grown overnight in YNB + 2% galactose, then 1:10 serial dilutions were prepared (initial dilution OD600 = 1.0). Strains were spotted onto the indicated media. Top row: comparing two carbon sources as indicated (both present at 2%). Bottom row: all YNB + 2% glucose plates containing the indicated quorum sensing molecules at 500 μM. Two biological replicates of tps1Δ were included for each strain background. Notably, the W303 wild type strain appears unable to grow in the presence of 500 μM farnesol. Strains used in this figure: DBY12000, DBY12383, DBY15117, DBY15121. (PDF) [file pone.0233779.s006.pdf]

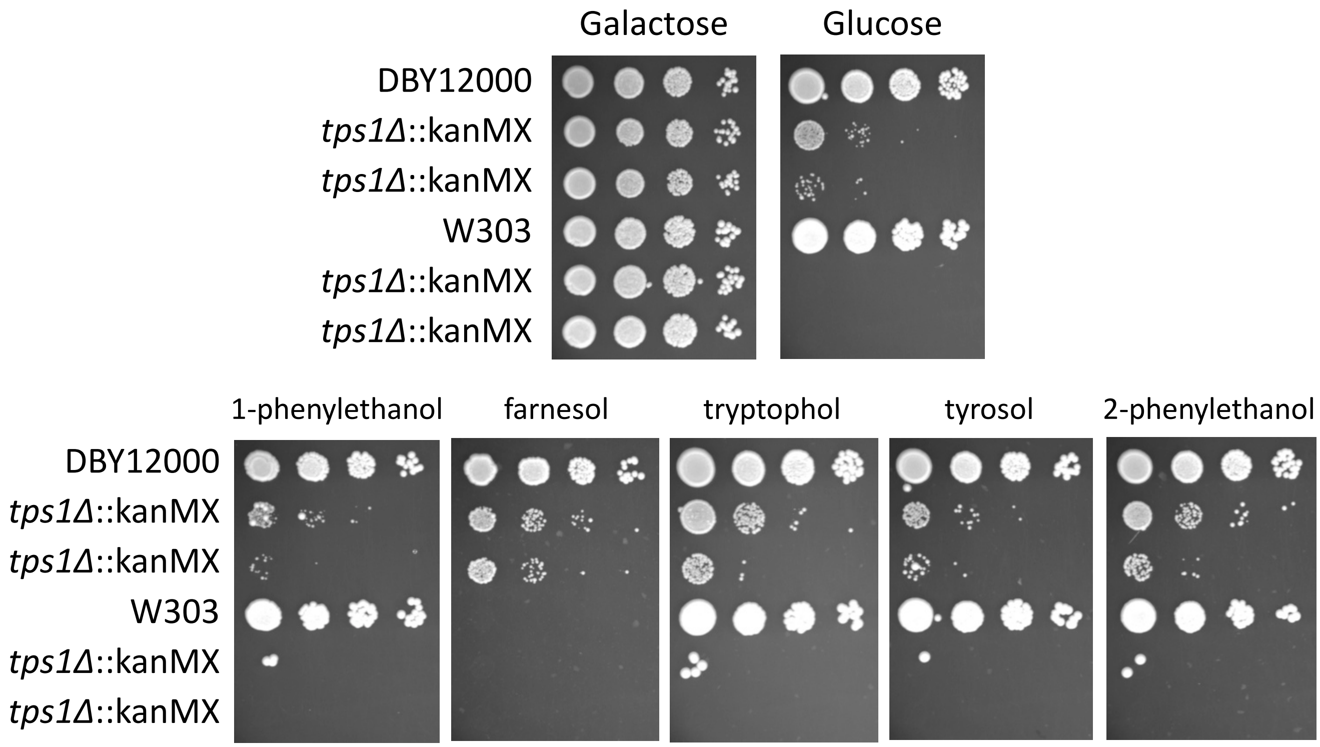


**Supplemental Figure 3. Yeast quorum sensing molecules do not influence the *tps1Δ* persister-like state.** The indicated strains were grown overnight in YNB + 2% galactose, then 1:10 serial dilutions were prepared (initial dilution $OD_{600}$ = 1.0). Strains were spotted onto the indicated media. Top row: comparing two carbon sources as indicated (both present at 2%). Bottom row: all YNB + 2% glucose plates containing the indicated quorum sensing molecules at 500 µM. Two biological replicates of *tps1Δ* were included for each strain background. Notably, the W303 wild type strain appears unable to grow in the presence of 500 µM farnesol. Strains used in this figure: DBY12000, DBY12383, DBY15117, DBY15121.
