# Supplementary material for: A tps1Δ persister-like state in Saccharomyces cerevisiae is regulated by MKT1
Source: PLoS One. 2020 May 29;15(5):e0233779. doi: 10.1371/journal.pone.0233779 (PMC7259636; doi:10.1371/journal.pone.0233779)
Supplement: S4 Fig — A. The indicated strains were grown overnight in YNB + 2% galactose, then 1:10 serial dilutions were prepared (initial dilution OD600 = 1.0). Strains were spotted onto the indicated media containing carbon sources at 2%. Three biological replicates of tps1Δ were included for each strain background. B. The indicated strains were grown overnight in YNB + 2% galactose, then 1:10 serial dilutions were prepared (initial dilution OD600 = 1.0). Strains were spotted onto the indicated media containing various nitrogen sources. Usable nitrogen was present at 76 mM (the amount in typical minimal medium). 2–3 biological replicates of tps1Δ were included for each strain background. All plates were incubated for 3 days at 30°C before photographing. Strains used in this figure: DBY12000, DBY12383, DBY15117, DBY15121, DBY12118. (PDF) [file pone.0233779.s007.pdf]

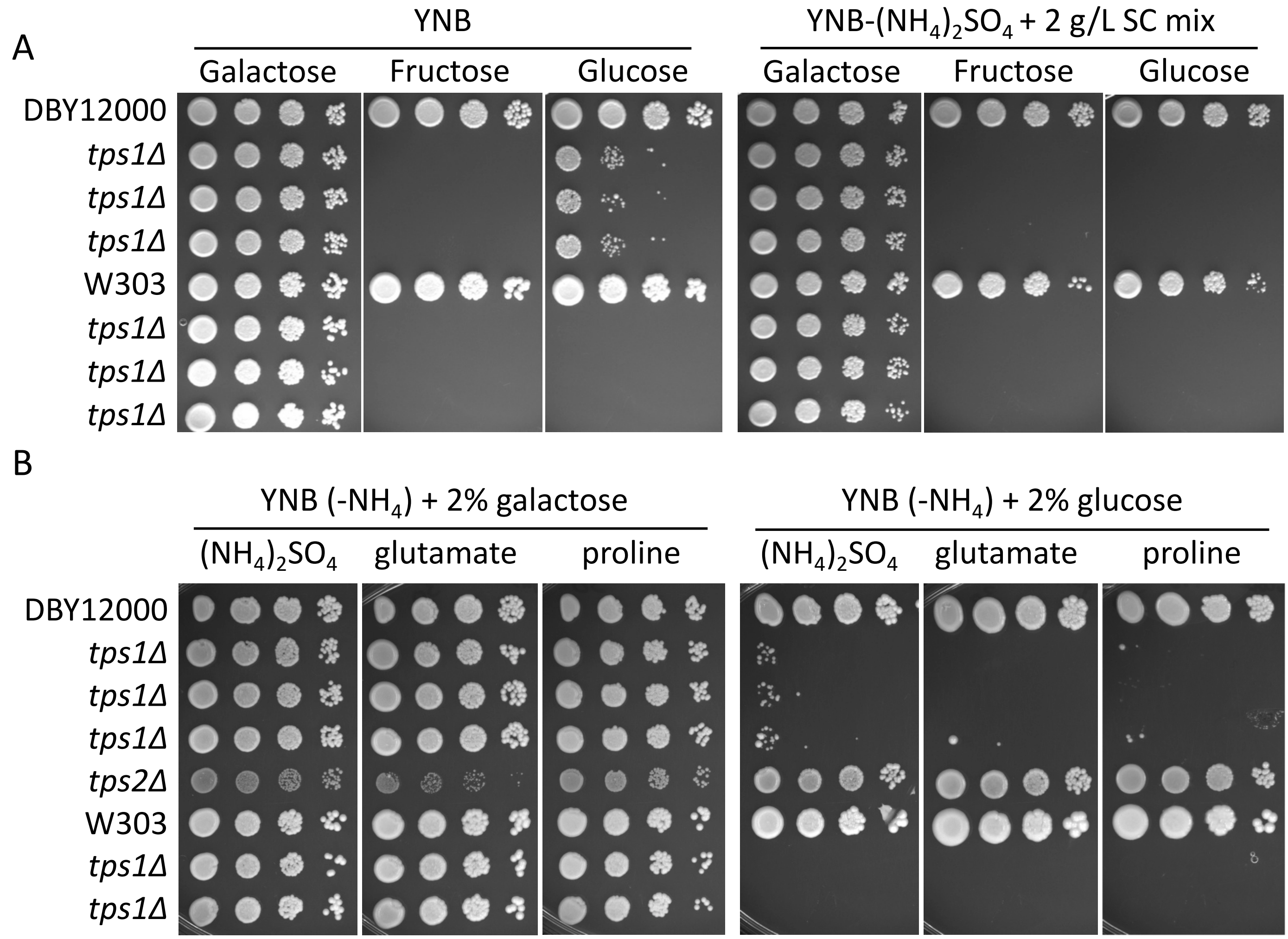

Supplemental Figure 4. The *tps1Δ* persister-like state is dependent on ammonia as a nitrogen source. A. The indicated strains were grown overnight in YNB + 2% galactose, then 1:10 serial dilutions were prepared (initial dilution OD_{600} = 1.0). Strains were spotted onto the indicated media containing carbon sources at 2%. Three biological replicates of *tps1Δ* were included for each strain background. **B**. The indicated strains were grown overnight in YNB + 2% galactose, then 1:10 serial dilutions were prepared (initial dilution OD_{600} = 1.0). Strains were spotted onto the indicated media containing various nitrogen sources. Usable nitrogen was present at 76 mM (the amount in typical minimal medium). 2-3 biological replicates of *tps1Δ* were included for each strain background. All plates were incubated for 3 days at 30°C before photographing. Strains used in this figure: DBY12000, DBY12383, DBY15117, DBY15121, DBY12118.
