# Supplementary material for: A tps1Δ persister-like state in Saccharomyces cerevisiae is regulated by MKT1
Source: PLoS One. 2020 May 29;15(5):e0233779. doi: 10.1371/journal.pone.0233779 (PMC7259636; doi:10.1371/journal.pone.0233779)
Supplement: S5 Fig — Strains were grown to early log phase in YNB + 2% galactose before addition of glucose or fructose to 2% as indicated. Cells were sampled at 0, 2.5, 5, 10, 15, 30, 60, and 120 minutes for RNA preparation. RNA-seq libraries and sequencing were performed as described in Materials and Methods. Data was analyzed as described in Materials and Methods. A—Heat- map showing wild type only. Indicated genes are *YDR524W-C (gene of unknown function) and **RPL41B (ribosomal 60S subunit L41B). B—Heat-map showing wild type and tps1Δ. In both A and B, individual clusters are indicated to the right of the heat-map, along with the Pearson correlation for each cluster shown parenthetically. C—Significantly enriched GO terms from highlighted clusters in panels A and B. GO terms were identified by searching using the SGD (www.yeastgenome.org) GO Slim Mapper tool, and examining Process (P), Function (F), Component (C), and Macromolecular Complex (M) data sets. Listed are terms manually curated as highly significant. Strains used in this figure: DBY12000, DBY12383. (PDF) [file pone.0233779.s008.pdf]

A

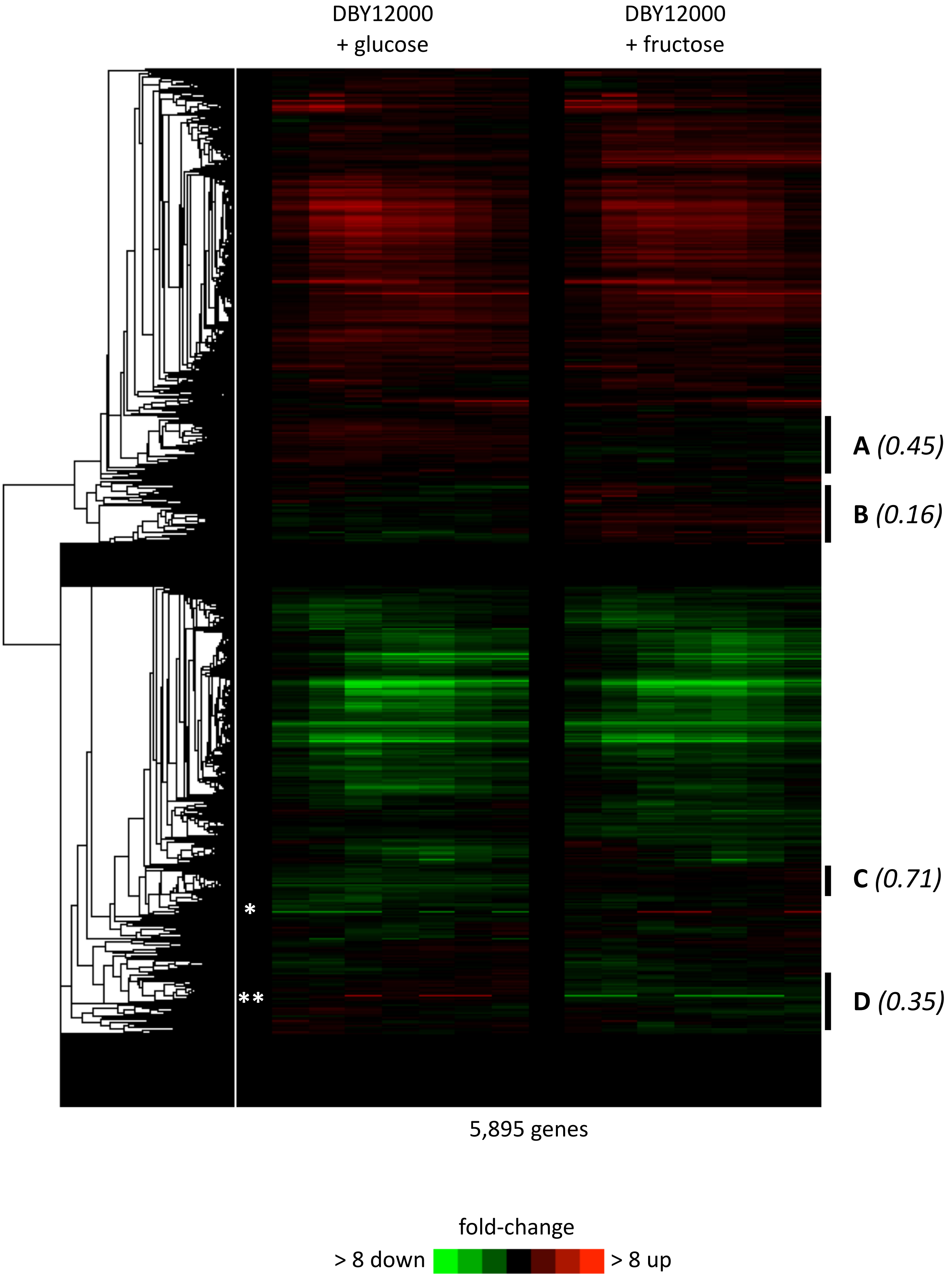
DBY12000
+ glucose
DBY12000
+ fructose
A (0.45)
B (0.16)
C (0.71)
D (0.35)
*
**
5,895 genes
fold-change
> 8 down
> 8 up

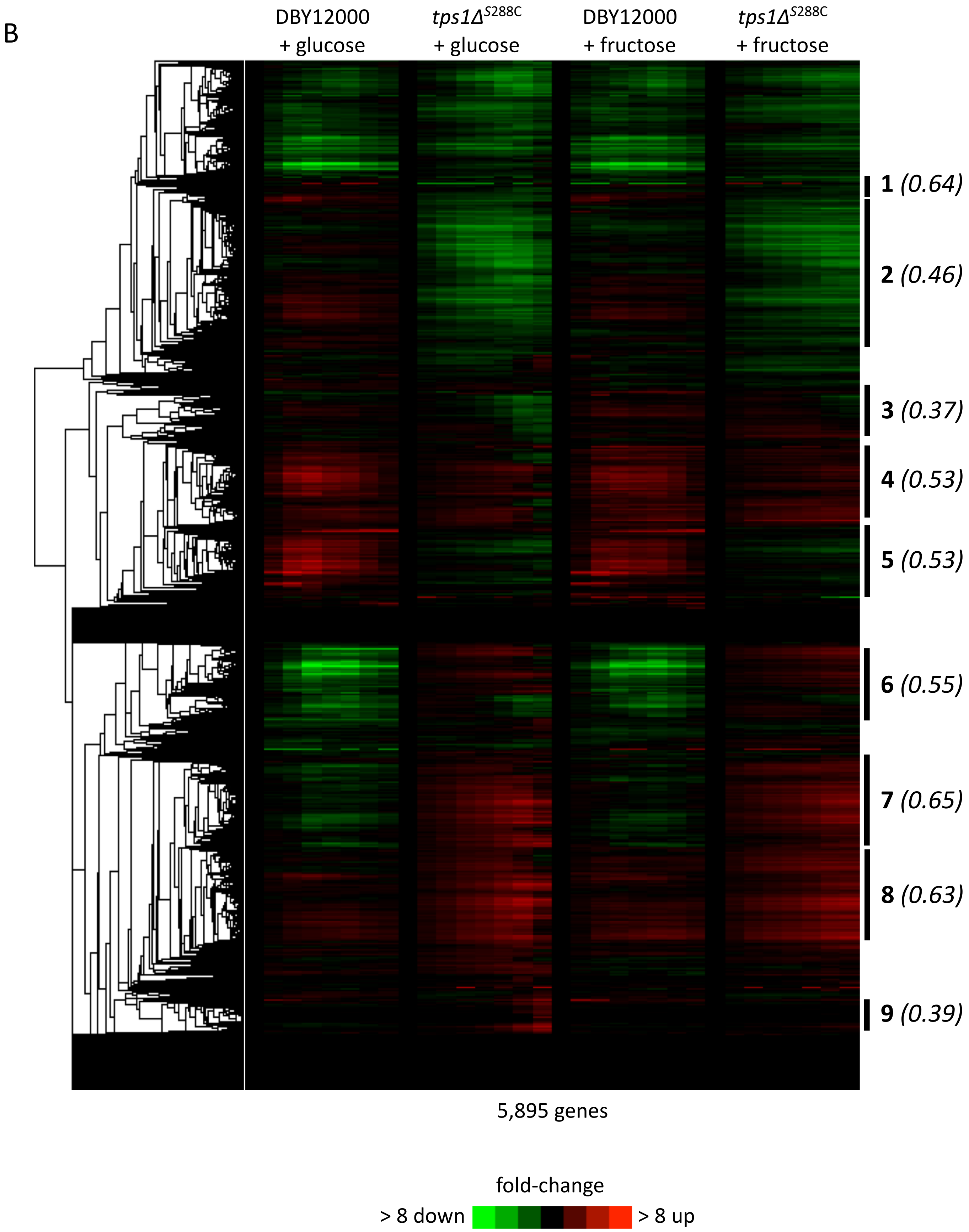
B
DBY12000
+ glucose
tps1Δ S288C
+ glucose
DBY12000
+ fructose
tps1Δ S288C
+ fructose
1 (0.64)
2 (0.46)
3 (0.37)
4 (0.53)
5 (0.53)
6 (0.55)
7 (0.65)
8 (0.63)
9 (0.39)
5,895 genes
fold-change
> 8 down
> 8 up

C

Cluster	Significantly enriched GO terms
**A**	chromatin (M), transcription factor complex (M), RNAPII holoenzyme (M), nucleus (C), chromosome (C), DNA binding (F), transcription factor activity (F), transcription from RNAPII promoter (P), mitotic cell cycle (P), chromatin organization (P), DNA repair (P), organelle fission (P)
**B**	endoplasmic reticulum (C), vacuoule (C), cytoplasmic vesicle (C), lipid metabolism (P), Golgi vesicle tranport (P)
**C**	endomembrane system (C), vacuole (C), endoplasmic reticulum (C), transmembrane transporter activity (F), lipid metabolism (P), transmembrane transport (P), protein glycosylation (P), Golgi vesicle transport (P)
**D**	chromatin (M), transcription factor complex (M), chromosome (C), cytoplasmic vesicle (C), DNA binding (F), transcription factor activity (F), transcription from RNAPII (P), mitotic cell cycle (P), chromatin organization (P)
**1**	transcription from RNAPII (P), signaling (P), response to chemical (P)
**2**	DNA binding (F), kinase activity (F), enzyme regulator activity (F), transcription from RNAPII (P), mitotic cell cycle (P), chromatin organization (P), organelle fission (P)
**3**	membrane (C), endoplasmic reticulum (C), ion transport (P), lipid metabolism (P), transmembrane transport (P), cell wall (P), transferase activity (F), hydrolase activity (F), glycosyl transferase activity (F)
**4**	ribonucleoprotein complex (M), ribosome (M), nucleus (C), nucleolus (C), structural constituent of ribosome (F), rRNA processing (P), cytoplasmic translation (P), ribosomal biogenesis (P)
**5**	ribonucleoprotein complex (M), ribosome (M), nucleus (C), nucleolus (C), ATPase activity (F), mRNA binding (F), helixase activity (F), rRNA processing (P), ribosomal biogenesis (P)
**6**	mitochondrial ribosome (M), mitochondrion (C), oxidoreductase activity (F), transmembrane transport activity (F), mitochondrion organization (P), generation of precursor metabolites and energy (P), protein complex biogenesis (P), nucleobase metabolism (P)
**7**	proteasome (M), oxidoreductase activity (F), protein complex biogenesis (P), proteolysis (P), lipid metabolism (P), cytoskeletal organization (P)
**8**	cytoplasmic ribosome (M), ribosome (C), structural constituent of ribosome (F), cytoplasmic translation (P), cellular amino acid metabolism (P)
**9**	spindle pole body (M), centromere (M), microtubule (M), plamsa membrane (C), chromosome (C), cytoplasmic vesicle (C), ATPase activity (F), organelle fission (P), cell cycle (P), cellular response to DNA damage (P), chromosome segregation (P)

Supplemental Figure 5. The gene expression response of wild type and *tps1Δ* cells to glucose and fructose exhibits strain- and condition-dependent effects. Strains were grown to early log phase in YNB + 2% galactose before addition of glucose or fructose to 2% as indicated. Cells were sampled at 0, 2.5, 5, 10, 15, 30, 60, and 120 minutes for RNA preparation. RNA-seq libraries and sequencing were performed as described in Materials and Methods. Data was analyzed as described in Materials and Methods. **A** - Heat-map showing wild type only. Indicated genes are **YDR524W-C* (gene of unknown function) and **RPL41B (ribosomal 60S subunit L41B). **B** - Heat-map showing wild type and *tps1Δ*. In both A and B, individual clusters are indicated to the right of the heat-map, along with the Pearson correlation for each cluster shown parenthetically. **C** - Significantly enriched GO terms from highlighted clusters in panels A and B. GO terms were identified by searching using the SGD (www.yeastgenome.org) GO Slim Mapper tool, and examining Process (P), Function (F), Component (C), and Macromolecular Complex (M) data sets. Listed are terms manually curated as highly significant. Strains used in this figure: DBY12000, DBY12383.
