# Supplementary material for: A tps1Δ persister-like state in Saccharomyces cerevisiae is regulated by MKT1
Source: PLoS One. 2020 May 29;15(5):e0233779. doi: 10.1371/journal.pone.0233779 (PMC7259636; doi:10.1371/journal.pone.0233779)
Supplement: S6 Fig — Strains were grown overnight in minimal media (glucose-containing for tps2Δ, galactose-containing for tps1Δ), then 1:10 serial dilutions were prepared (initial dilution OD600 = 1.0) and spotted onto the indicated media and incubated at indicated temperatures for 3 days before photographing. Strains used in this figure: DBY12000, DBY12134, DBY12383, DBY12118, DBY12813. (PDF) [file pone.0233779.s009.pdf]

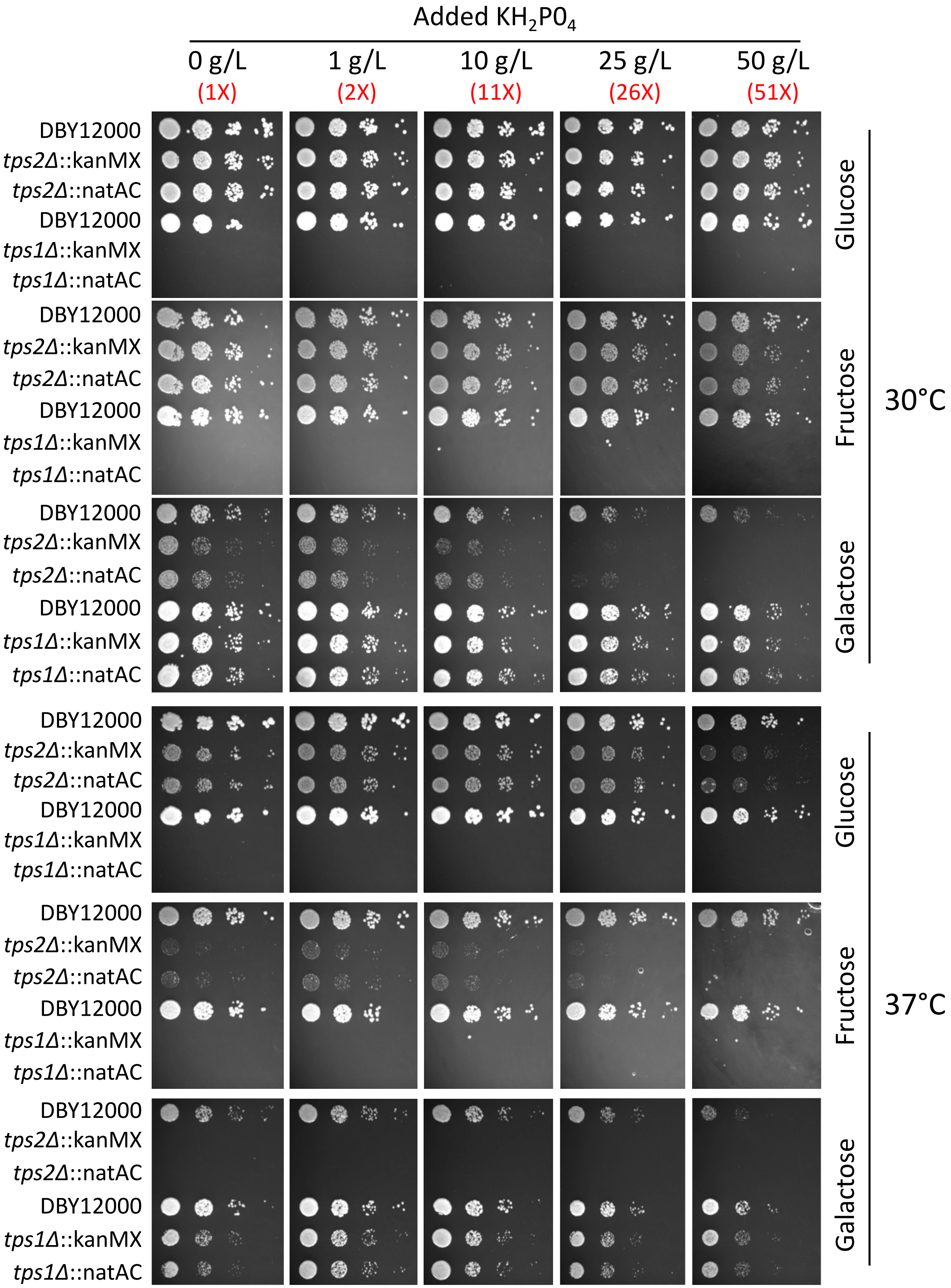

Supplemental Figure 6. Neither *tps1Δ* nor *tps2Δ* growth defects are restored by addition of exogenous phosphate to growth media. Strains were grown overnight in minimal media (glucose-containing for *tps2Δ*, galactose-containing for *tps1Δ*), then 1:10 serial dilutions were prepared (initial dilution OD_{600} = 1.0) and spotted onto the indicated media and incubated at indicated temperatures for 3 days before photographing. Strains used in this figure: DBY12000, DBY12134, DBY12383, DBY12118, DBY12813.
