# Supplementary material for: A tps1Δ persister-like state in Saccharomyces cerevisiae is regulated by MKT1
Source: PLoS One. 2020 May 29;15(5):e0233779. doi: 10.1371/journal.pone.0233779 (PMC7259636; doi:10.1371/journal.pone.0233779)
Supplement: S8 Fig — (PDF) [file pone.0233779.s011.pdf]

Supplemental Figure 8. Potential causative mutations on chromosome XIV between 440,000 and 490,000 kb (within coding regions)

Gene	Function	Changes[a]
PHO23	histone deacetylase complex component	F->L
tRNA-Leu	encodes leucine tRNA	see notes[b]
YNL095C	putative protein of unknown function	F->L, V->F, T->I, V->A, D->E
APP1	component of endocytic pathway	A->T
YNL092W	putative SAM-dependent methyltransferase	A->E
NST1	protein of unknown function	E->D, R->G
RHO2	involved in cell polarity and microtubule assembly	F->C
SNN1	subunit of endosomal maturation complex	I->M
MKT1	forms complex with Pbp1 in glucose-deprivation stress granules; allelic variation affects mitochondrial genome stability, drug resistance, and more	D->G, K->R
SAL1	ADP/ATP transporter active in mitochondria during fermentation	V-M, G->W, G->A, frameshift[c]
SWS2	putative mitochondrial ribosomal small subunit	F->L
TPM1	major isoform of tropomyosin; binds/stabilizes actin cables and filaments	H->Q
NIS1	protein localized to the bud neck during G2/M	Y->M, L->W, E->D, S->R
APJ1	hsp40 with a role in SUMO-mediated protein degradation	D->N
MKS1	pleiotropic negative transcriptional regulator; involved in Ras/cAMP signaling and nitrogen regulation	H->Y, P->A, D->E, P->T, E->G
IMP4	component of the SSU processome	S->G

[a]amino acid changes in S288C-background compared to W303 background (S288C->W303)

[b]single base change present in S288C compared to W303

[c]frameshift mutation truncates S288C *SAL1*
