# Supplementary material for: A tps1Δ persister-like state in Saccharomyces cerevisiae is regulated by MKT1
Source: PLoS One. 2020 May 29;15(5):e0233779. doi: 10.1371/journal.pone.0233779 (PMC7259636; doi:10.1371/journal.pone.0233779)
Supplement: S9 Fig — The indicated strains, including URA3+ segregants from DBY12796 (MKS1 test) and DBY12795 (SAL1 test) were grown overnight in YNB + 2% galactose. Next, 1:10 serial dilutions were prepared (initial dilution OD600 = 1.0) and strains were spotted onto the indicated media, then incubated for 3 days at 30°C. (PDF) [file pone.0233779.s012.pdf]

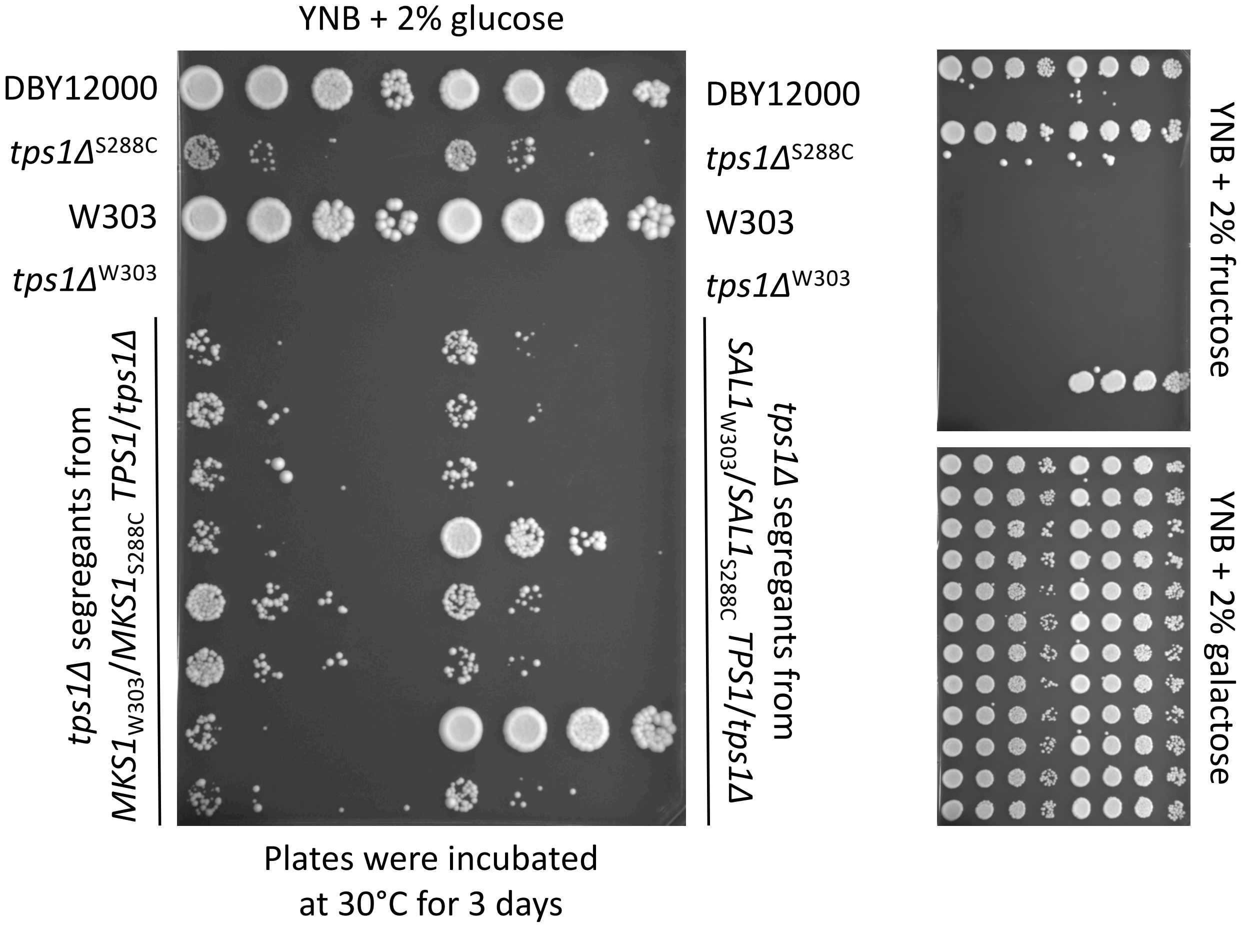

Supplemental Figure 9. Mutations in *MKS1* or *SAL1* in S288C compared to W303 are not responsible for *tps1Δ* persister-like activity. The indicated strains, including *URA3*$^{+}$ segregants from DBY12796 (*MKS1* test) and DBY12795 (*SAL1* test) were grown overnight in YNB + 2% galactose. Next, 1:10 serial dilutions were prepared (initial dilution OD_{600} = 1.0) and strains were spotted onto the indicated media, then incubated for 3 days at 30°C.
