# Supplementary material for: A tps1Δ persister-like state in Saccharomyces cerevisiae is regulated by MKT1
Source: PLoS One. 2020 May 29;15(5):e0233779. doi: 10.1371/journal.pone.0233779 (PMC7259636; doi:10.1371/journal.pone.0233779)
Supplement: S10 Fig — The initial dilution had an OD600 of 1.0. Listed carbon sources were present at 2%. Plates were incubated at 30°Cfor 3 days. Cells from each strain shown were grown overnight in YP + 2% Galactose for genomic DNA preparation, MKT1 PCR amplification, and sequencing. The identified allele present is indicated for each strain: red for the W303 allele, or blue for the S288C allele. (PDF) [file pone.0233779.s013.pdf]

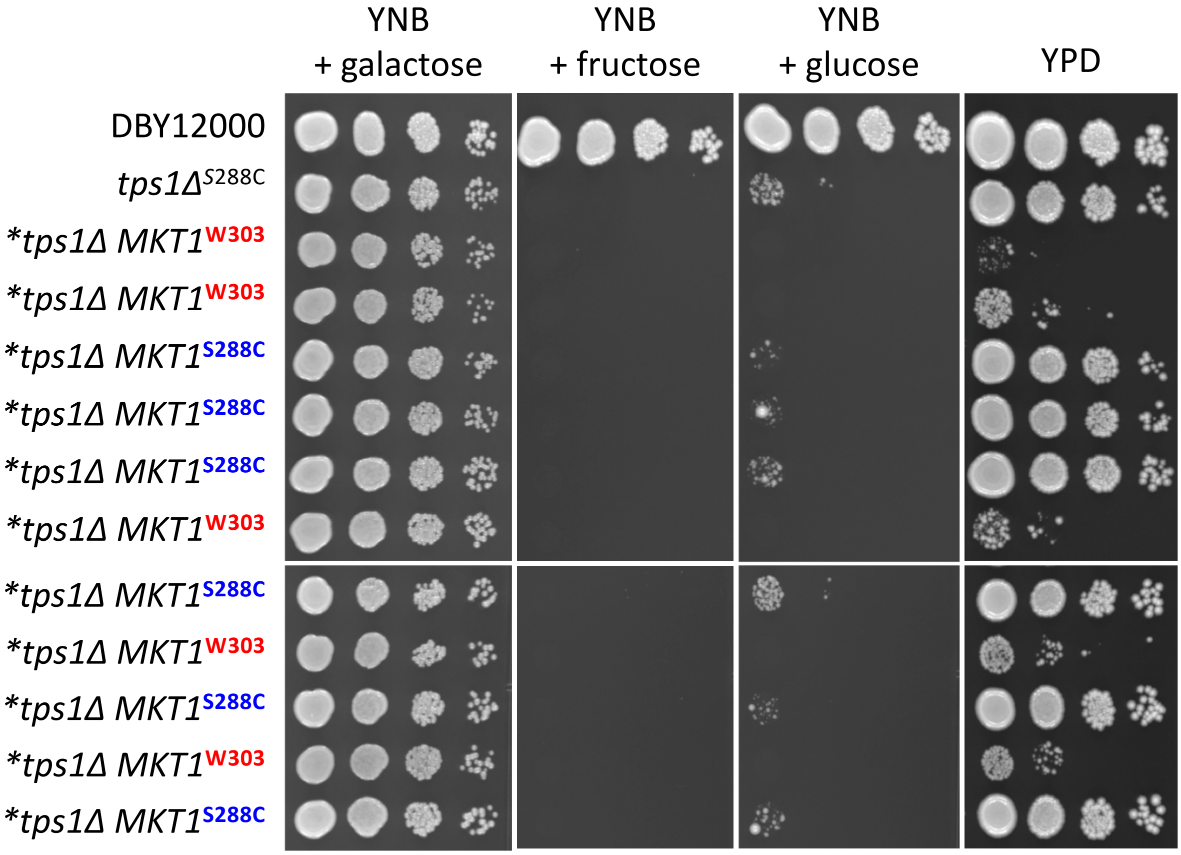

Supplemental Figure 10. *MKT1* regulates the *tps1Δ* persister-like state . *URA3*⁺ *tps1Δ* segregants from DBY12821 (along with the indicated wild type and *tps1Δ* controls) were grown overnight in YNB + 2% galactose liquid before 10-fold serial dilutions were prepared and spotted onto the indicated media. The initial dilution had an OD_{600} of 1.0. Listed carbon sources were present at 2%. Plates were incubated at 30°Cfor 3 days. Cells from each strain shown were grown overnight in YP + 2% Galactose for genomic DNA preparation, *MKT1* PCR amplification, and sequencing. The identified allele present is indicated for each strain: red for the W303 allele, or blue for the S288C allele.
