# Supplementary material for: A tps1Δ persister-like state in Saccharomyces cerevisiae is regulated by MKT1
Source: PLoS One. 2020 May 29;15(5):e0233779. doi: 10.1371/journal.pone.0233779 (PMC7259636; doi:10.1371/journal.pone.0233779)
Supplement: S11 Fig — Indicated strains were grown overnight in YNB + 2% galactose liquid before 10-fold serial dilutions were prepared and spotted onto the indicated media. The initial dilution had an OD600 of 1.0. Listed carbon sources were present at 2%. Plates were incubated at 30°C for 3 days. Strains used in this figure: DBY15039, PGY185. (PDF) [file pone.0233779.s014.pdf]

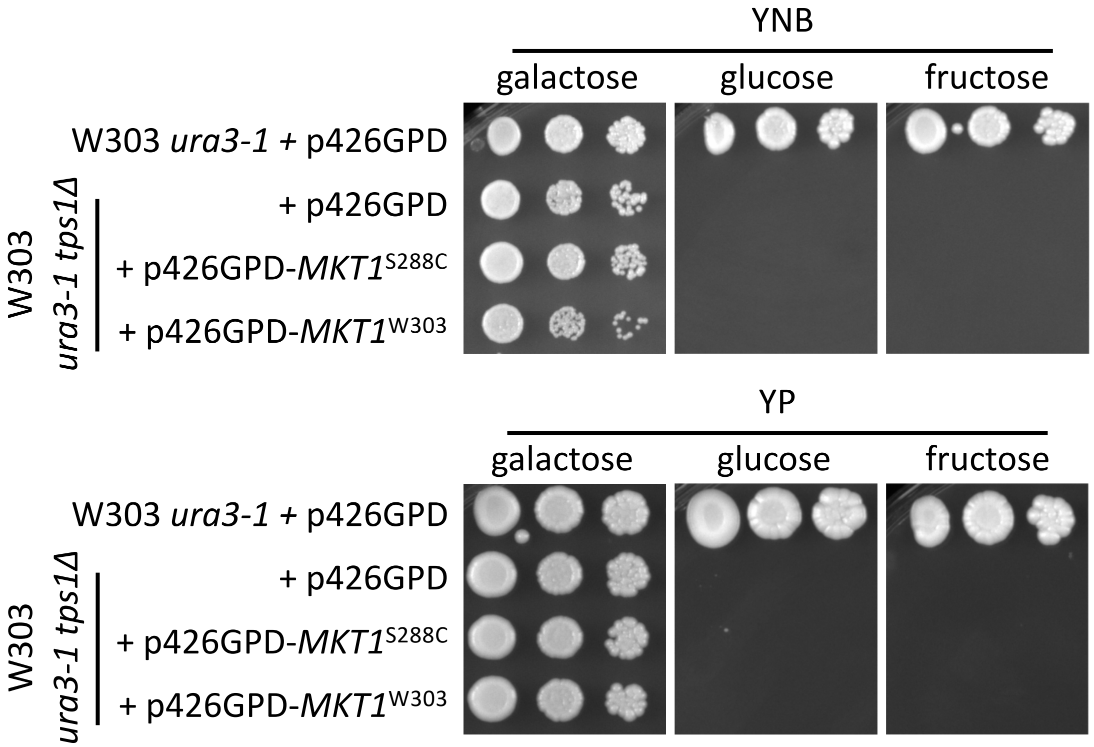

Supplemental Figure 11. Overexpression of *MKT1*^{S288C} in W303 tps1 does not induce persister-like state, suggesting the *MKT1*^{S288C} allele is recessive. Indicated strains were grown overnight in YNB + 2% galactose liquid before 10-fold serial dilutions were prepared and spotted onto the indicated media. The initial dilution had an OD_{600} of 1.0. Listed carbon sources were present at 2%. Plates were incubated at 30°C for 3 days. Strains used in this figure: DBY15039, PGY185.
