# Supplementary material for: A tps1Δ persister-like state in Saccharomyces cerevisiae is regulated by MKT1
Source: PLoS One. 2020 May 29;15(5):e0233779. doi: 10.1371/journal.pone.0233779 (PMC7259636; doi:10.1371/journal.pone.0233779)
Supplement: S7 Fig — Segregants from DBY12689 (A) and DBY12688 (B) were spotted onto the indicated minimal media and grown 3 days at 30°C (3 x 10-fold serial dilutions; initial dilution OD600 = 1.0). C. Strains with tps1Δ persister- like activity (+) and strains without (-) were pooled from both sets of segregants and sequenced. Shown is a IGV browsing window zoomed into chromosome XIV. Because the reference for sequence alignment was an S288C derivative, all W303-specific mutations appear as colored vertical bars. Note that strains that fail to exhibit tps1Δ persister-like activity (-) all contain the W303 genetic material for this region, while the opposite is true for those segregants that do exhibit tps1Δ persister-like activity (+). (PDF) [file pone.0233779.s010.pdf]

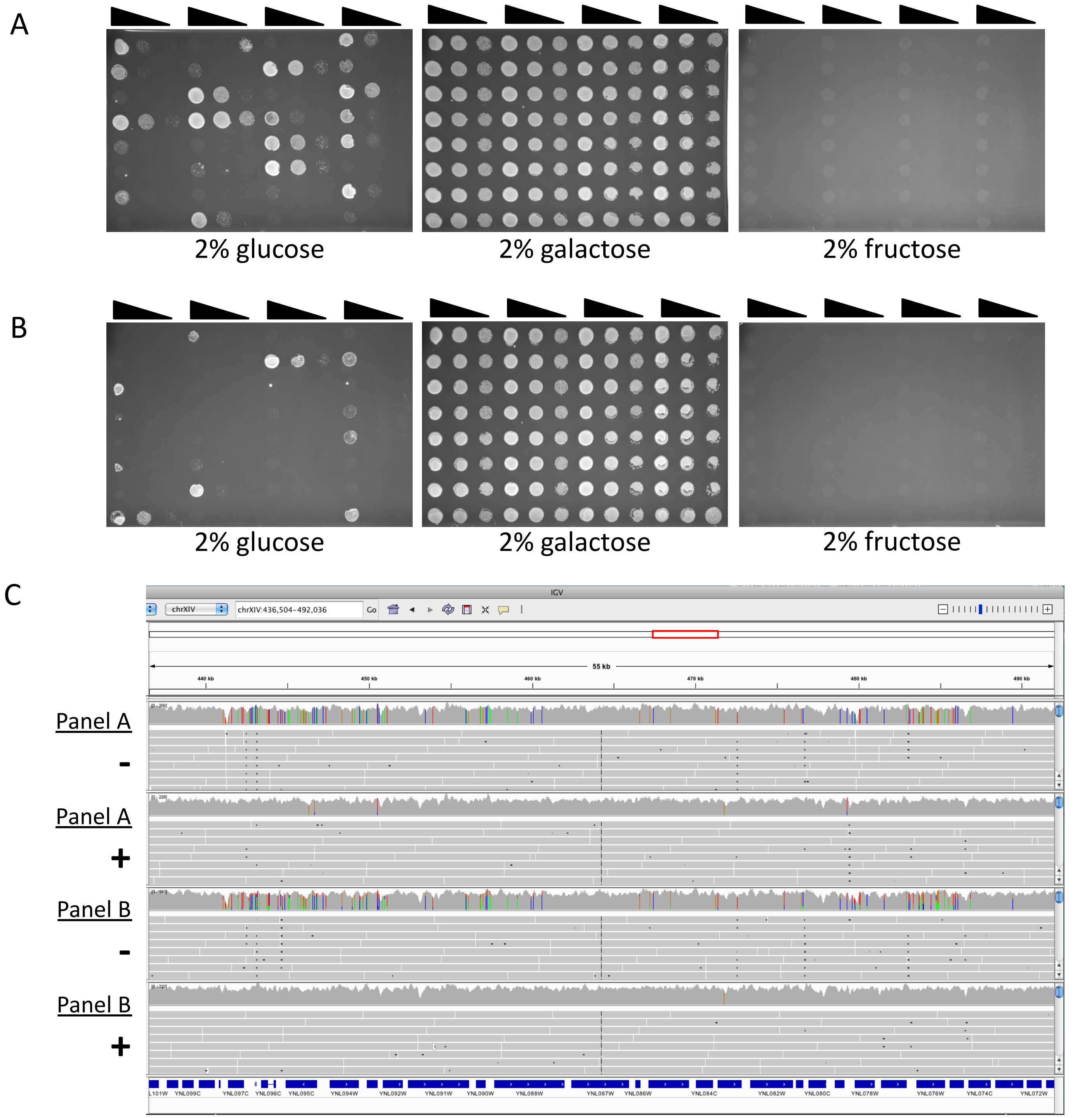

Supplemental Figure 7. Segregation of *tps1Δ* persister-like activity to a region on chromosome XIV. Segregants from DBY12689 (**A**) and DBY12688 (**B**) were spotted onto the indicated minimal media and grown 3 days at 30°C (3 x 10-fold serial dilutions; initial dilution OD_{600} = 1.0). **C**. Strains with *tps1Δ* persister-like activity (**+**) and strains without (**-**) were pooled from both sets of segregants and sequenced. Shown is a IGV browsing window zoomed into chromosome XIV. Because the reference for sequence alignment was an S288C derivative, all W303-specific mutations appear as colored vertical bars. Note that strains that fail to exhibit *tps1Δ* persister-like activity (**-**) all contain the W303 genetic material for this region, while the opposite is true for those segregants that do exhibit *tps1Δ* persister-like activity (**+**).
